# SENP3 Drives Abdominal Aortic Aneurysm Development by Regulating Ferroptosis via De‐SUMOylation of CTH

**DOI:** 10.1002/advs.202414500

**Published:** 2025-02-28

**Authors:** Long Chen, Zhaohua Cai, Danrui Xiao, Yiping Shi, Qingqing Xiao, Min Liang, Yangjing Jiang, Yijie Huang, Feng Liang, Guo Zhou, Fei Zhuang, Xia Wang, Huanhuan Huo, Liang Fang, Qin Shao, Ben He

**Affiliations:** ^1^ Department of Cardiology Shanghai Chest Hospital Shanghai Jiao Tong University School of Medicine Shanghai 200030 China

**Keywords:** abdominal aortic aneurysm, CTH, ferroptosis, SENP3, SUMOylation

## Abstract

Abdominal aortic aneurysm (AAA) is a high‐risk inflammatory disorder. SENP3, a SUMO2/3‐specific protease, is closely involved in the development of cancer. In this study, the aim is to explore the role of SENP3 in macrophages in AAA. It is found that the protein expression of SENP3 is significantly upregulated in both human and murine AAA specimens. SENP3 expression is negatively regulated by the E3 ubiquitin ligase STUB1/CHIP. Furthermore, myeloid‐specific SENP3 knockout inhibited AAA formation in both AngII‐ and CaCl_2_‐induced mouse models. SENP3 deficiency repressed AAA lesion macrophage infiltration and inflammatory response. Mechanistic studies identified Cystathionine Gamma–Lyase (CTH), a critical enzyme involved in hydrogen sulfide production, as a target protein of SENP3 that mediated the exacerbating effects of SENP3 on ferroptosis and inflammatory programs in macrophages. SUMO‐3 modification at Lysine 361 promoted CTH protein stability, whereas de‐SUMOylation by SENP3 facilitated its proteasome‐dependent degradation. Most importantly, it is found that CTH inhibitor counteracted the protective effect of SENP3 deficiency on AAA. Additionally, supplementation with ATB346, a novel H_2_S‐donating naproxen derivative, prevented AAA development in mice. These studies suggest that SENP3‐mediated CTH deSUMOylation regulates macrophage ferroptosis and AAA development. The SENP3/CTH axis is therefore an important therapeutic target for aortic aneurysmal diseases.

## Graphical abstract

In this study, it is proposed that SENP3 accumulates in macrophages of aneurysmal aortic tissues, which promotes macrophage ferroptosis and inflammation via regulation of CTH de‐SUMOylation.

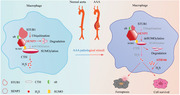

## 1 Introduction

Abdominal aortic aneurysm (AAA) is an age‐related disease, characterized by progressive segmental dilation of the abdominal aorta of 50% or more compared to the normal diameter, with an ≈80% mortality rate upon rupture.^[^ ^1^, ^2^ ^]^ Currently, endovascular stenting and open surgery are the mainstays of treatment for AAA, and no medication has been approved by the U.S. Food and Drug Administration (FDA) to induce regression or slow AAA growth and restrain the risk of rupture.^[^ ^3^ ^]^ Thus, clarifying the potential molecular mechanisms and mining novel and efficient therapeutic targets are crucial.

Inflammation is the hallmark of AAA with macrophages being predominant. In clinical samples and animal models, macrophages play an essential and distinct role in AAA development and progression to rupture.^[^ ^4^ ^]^ During vascular remodeling, monocytes are recruited to aneurysm tissues and differentiate into macrophages that participate in inflammatory cytokine expression, proteolytic enzyme production, extracellular matrix degradation, and pathological aortic dilation, together with specialized tissue‐resident macrophages.^[^ ^5^ ^]^ However, the critical pathways through which macrophages facilitate inflammation during AAA formation remain insufficiently elucidated. Ferroptosis is an iron‐dependent form of cell death, which is characterized by the production of reactive oxygen species (ROS) from accumulated iron and lipid peroxidation.^[^ ^6^ ^]^ Previous studies have indicated that ferroptosis facilitates the progression and associated inflammation of AAA.^[^ ^7^, ^8^, ^9^ ^]^ Macrophage ferroptosis is therefore a potential therapeutic target for prevention of AAA development.

Post‐translational modifications (PTMs), such as ubiquitination, phosphorylation, glycosylation, and SUMOylation, play important roles in modulating protein functions. Among them, SUMOylation entails the conjugation of Small Ubiquitin‐like Modifier (SUMO) to lysine residue(s) on target proteins, thereby influencing the subcellular localization, protein stability, and protein‐protein interactions. Notably, SUMOylation can be reversed by Sentrin/SUMO‐specific proteases (SENPs), releasing the SUMO molecule(s) for the next cycle.^[^ ^10^ ^]^ We have previously demonstrated that SENP3, one of the members of SENPs family, plays critical roles in cardiovascular and metabolic diseases via its de‐SUMOylation of protein substrates.^[^ ^11^, ^12^ ^]^ SENP3 promotes vascular smooth muscle cell (VSMC) proliferation and migration in vascular remodeling via de‐SUMOylation of β‐catenin.^[^ ^11^ ^]^ SENP3 de‐SUMOylates YAP1 and regulates macrophage inflammation during diet and age‑induced obesity.^[^ ^12^ ^]^ However, the role of SENP3 in aortic aneurysmal diseases has never been investigated.

In this study, we aimed to investigate whether SENP3 mediates the pathophysiology of AAA, and to explore possible underlying molecular mechanisms. We found that SENP3 contributes to AAA formation and macrophage ferroptosis by targeting cystathionine‐γ‐lyase (CTH), a hydrogen sulfide (H_2_S)‐synthesizing enzyme. Moreover, ATB346, a novel H_2_S‐releasing drug, ameliorates the vascular inflammation and restricted AAA formation.

## 2 Result

### 2.1 The Protein Expression of SENP3 is Markedly Upregulated in Human and Murine AAA Tissues

**Figure 1:**
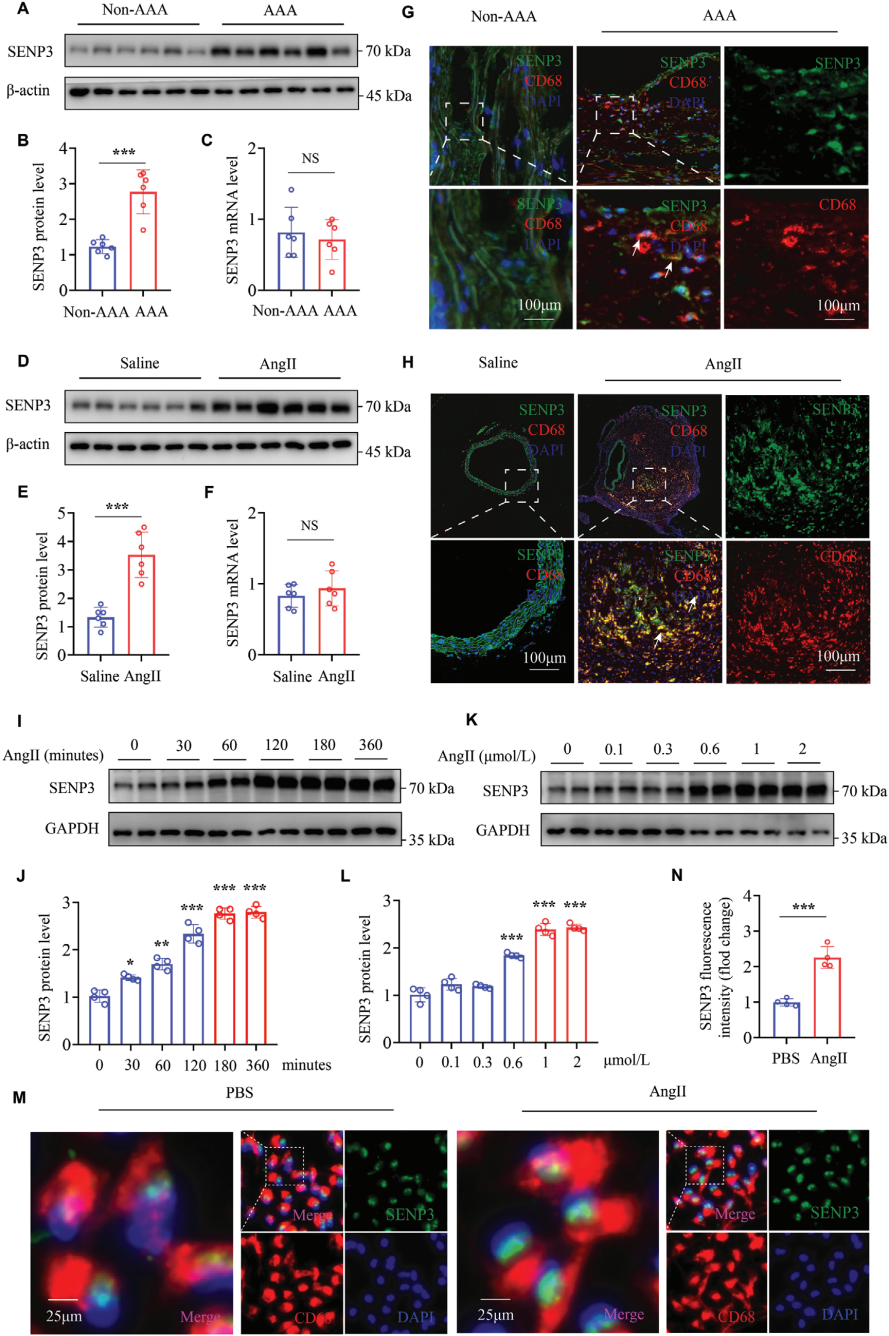
The protein expression of SENP3 is significantly upregulated in human and murine AAA tissues. A–C) SENP3 protein and mRNA levels were determined by western blot (A,B) and real‐time quantitative polymerase chain reaction (RT‐qPCR; C) in human abdominal aortic aneurysm (AAA) samples and adjacent nonaneurysmal control samples (non‐AAA) (*n* = 6 per group). D–F) SENP3 protein and mRNA levels were determined by western blot (D,E) and RT‐qPCR (F) in abdominal aortic samples of *ApoE^−/−^* mice infused with angiotensin II (AngII) or saline for 28 days (*n* = 6 per group). G) Representative images of dual immunofluorescence staining of SENP3 (green) and CD68 (red) in human AAA samples and adjacent nonaneurysmal control samples. Where indicated, nuclei were counterstained with 4′,6‐diamidino‐2‐phenylindole (DAPI; blue). Scale bar: 100 µm. H) Representative images of dual immunofluorescence staining for SENP3 (green) and CD68 (red) in abdominal aortic samples of *ApoE^−/−^* mice infused with AngII or saline for 28 days. Scale bar: 100 µm. I,J) SENP3 protein levels were determined by western blot in BMDMs after stimulation with AngII (1 µmol L^−1^) for the indicated time periods (*n* = 4 per group). K,L) SENP3 protein levels were determined by western blot in bone marrow‐derived macrophages (BMDMs) after stimulation with indicated concentrations of AngII for 180 min (*n* = 4 per group). M,N) Representative images of dual immunofluorescence staining for SENP3 (green) and CD68 (red) in BMDMs stimulated with AngII (1 µmol L^−1^) for 180 min (M). Where indicated, nuclei were counterstained with DAPI (blue). Scale bar: 25 µm. Quantification of fluorescence intensity of SENP3 (N; *n* = 4 for each group). Data represent mean ± SEM. *p* values were determined using the student's *t*‐test (B,C,E,F,N) and Welch ANOVA test (J,L). For all panels, *p < 0.05; **p < 0.01; ***p < 0.001; ns: not significant (*p* > 0.05).

To explore whether SENP3 is involved in the pathogenesis of AAA, we first examined the expression of SENP3 in AAA tissues. SENP3 protein levels were strongly increased, whereas the mRNA levels remained unaltered in human AAA tissues (**Figure** **1**A–C). Consistently, SENP3 protein levels were markedly increased in the aneurysmal aortas from AngII‐infused *ApoE^−/−^* mice (Figure 1D–F) and CaCl_2_‐treated C57BL/6J mice (Figure S1A,B, Supporting Information). Immunofluorescence staining revealed a heightened presence of infiltrating macrophages in both human and murine AAA tissues, with high expression of SENP3 within these macrophages (Figure 1G,H; Figure S1C, Supporting Information).

To further investigate the role of SENP3 in macrophages in AAA, mouse bone marrow‐derived macrophages (BMDMs) and human THP‐1‐derived macrophages were treated with AngII in vitro. AngII markedly increased SENP3 protein expression in a time‐ and dose‐dependent manner (Figure 1I–L; Figure S2, Supporting Information). However, no significant change in SENP3 mRNA level was observed after AngII stimulation (Figure S3A,B, Supporting Information). Immunofluorescence staining further confirmed the upregulation of SENP3 protein levels in BMDMs after AngII stimulation (Figure 1M,N). Moreover, we found that AngII increased intracellular ROS levels in BMDMs and triggered SENP3 accumulation, which was completely abolished by ROS scavengers phenyl N‐t‐butylnitrone (PBN) and *N*‐acetyl cysteine (NAC) (Figure S4A,B, Supporting Information), consistent with previous findings suggesting that SENP3 is a redox‐sensitive protein.^[^ ^11^, ^13^ ^]^

Altogether, these results suggest that SENP3 is upregulated in human and murine AAA via an ROS‐dependent manner.

### 2.2 SENP3 Expression is Negatively Regulated by the E3 Ubiquitin Ligase STUB1/CHIP

Previous studies have reported that STUB1/CHIP, an essential E3 ubiquitin ligase, facilitates the degradation of SENP3 under non‐stress conditions in tumor cells.^[^ ^13^ ^]^ Recently, STUB1/CHIP has been implicated in the pathogenesis of atherosclerosis via control of SIRT6 stability in VSMCs.^[^ ^14^ ^]^ To explore the molecular mechanism by which SENP3 expression is regulated in AAA, we interrogated the expression profile of STUB1 in macrophages in AAA. We observed that the expression of STUB1 was notably decreased in BMDMs after AngII stimulation, in parallel with the upregulation of SENP3 (Figure S5A, Supporting Information). Immunofluorescence staining further confirms the downregulation of STUB1 in BMDMs after AngII stimulation (Figure S5B, Supporting Information). The protein expression levels of STUB1 were further assessed in human and murine AAA specimens. Western blotting analysis suggested the dramatical downregulation of STUB1 in human AAA tissues (Figure S5C, Supporting Information) and aneurysmal aortas from AngII‐infused *ApoE^−/−^* mice (Figure S5D, Supporting Information).

We next investigated the effects of STUB1 knockdown and overexpression on SENP3 expression. We noted that small interfering RNA (siRNA)‐mediated STUB1 knockdown led to a robust increase of SENP3 in BMDMs (Figure S5E, Supporting Information), whereas STUB1 overexpression resulted in a strong reduction of SENP3 protein levels (Figure S5F, Supporting Information). Moreover, we found that STUB1‐mediated SENP3 elimination was abolished following treatment with the 26S proteasome inhibitor MG132 in BMDMs (Figure S5G, Supporting Information).

To further explore the role of STUB1 in regulation of SENP3 stability, a co‐immunoprecipitation assay was performed to validate their interaction. As expected, the interaction between STUB1 and SENP3 was demonstrated by immunoprecipitation assay in BMDMs (Figure S5H,I, Supporting Information). In addition, we found that overexpression of STUB1 triggered polyubiquitination of SENP3 (Figure S5J, Supporting Information). These collective data suggest that the E3 ubiquitin ligase STUB1 targets SENP3 for ubiquitination and degradation.

### 2.3 Myeloid‐Specific SENP3 Deficiency Blunts AAA Formation in Mice

**Figure 2:**
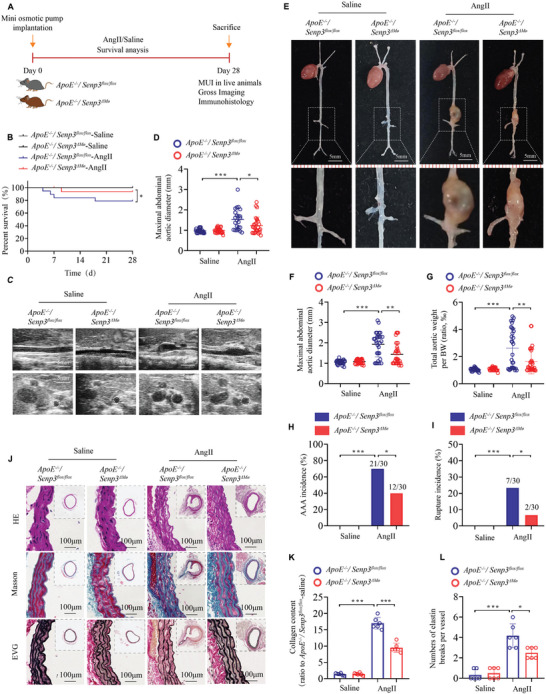
Myeloid‐specific SENP3 deficiency represses AngII‐induced AAA formation. A) Schematic protocol: *ApoE^−/−^;Senp3^flox/flox^* and *ApoE^−/−^;Senp3^△Mø^* mice were subcutaneously injected with saline or AngII via a mini osmotic pump for 28 days (*n* = 30 per group). B) Survival curves in the indicated groups (*n* = 30 per group). Survival data were analyzed by the Kaplan–Meier method and compared using the log‐rank test. C,D) Representative images and quantification of the maximal abdominal aortic diameter monitored by micro‐ultrasound imaging (MUI) in the indicated groups (*n* = 20–30 per group). Scale bar: 5 mm. E) Representative images of macroscopic features of AAA formation in the indicated groups. Scale bar: 5 mm. F,G) Quantification of the maximal abdominal aortic diameter measured by a digital vernier caliper and ratio of aortic weight to body weight (BW) in the indicated groups (*n* = 20–30 per group). H,I) The incidences of AngII‐induced AAA (H) and aortic rupture (I) in the indicated groups (*n* = 30 per group). J) Representative images of suprarenal aortic sections stained with hematoxylin and eosin (H&E), Masson Trichrome, and Van Gieson after saline or Ang II infusion (*n* = 6 per group). Scale bar: 100 µm. K,L) Quantification of relative collagen content (K) and numbers of elastin breaks per vessel (L) in each group. Data represent mean ± SEM. *p* values were determined using two‐way ANOVA followed by Bonferroni post‐hoc test (D,F,G,K,L) and Fisher exact test (H,I). For all panels, *p < 0.05; **p < 0.01; ***p < 0.001.

To explore the functional effects of SENP3 on the pathogenesis of AAA, *ApoE^−/−^;Senp3^flox/flox^* and *ApoE^−/−^;Senp3^△Mø^* mice were subcutaneously injected with AngII or saline via osmotic minipumps for 28 days (Figure S6, Supporting Information; **Figure** **2**A). *ApoE^−/−^;Senp3^△Mø^* mice displayed better survival than *ApoE^−/−^;Senp3^flox/flox^* mice after 28 days of AngII‐infusion (93.3% in *ApoE^−/−^;Senp3^△Mø^* mice compared with 76.7% in *ApoE^−/−^;Senp3^flox/flox^* mice; *p* < 0.05) (Figure 2B). To non‐invasively assess the aortic enlargement after AngII infusion in vivo, we performed micro‐ultrasound imaging and found that AngII‐infused *ApoE^−/−^;Senp3^△Mø^* mice showed significantly smaller maximal internal diameters compared with *ApoE^−/−^;Senp3^flox/flox^* mice (Figure 2C,D). Upon dissection, we further confirmed the decreased maximal abdominal aortic diameter and ratio of aortic weight to body weight in *ApoE^−/−^;Senp3^△Mø^* mice, compared to *ApoE^−/−^;Senp3^flox/flox^* mice (Figure 2E–G). Notably, myeloid‐specific SENP3 deficiency was associated with a significantly decreased incidence of AAA formation after AngII infusion (40% [12 of 30] of *ApoE^−/−^;Senp3^△Mø^* mice compared with 70% [21 of 30] of *ApoE^−/−^;Senp3^flox/flox^* mice; *p* < 0.05) (Figure 2H). Moreover, the incidence of aortic rupture in *ApoE^−/−^;Senp3^△Mø^* mice was also significantly decreased after AngII infusion compared with *ApoE^−/−^;Senp3^flox/flox^* mice (6.67% [2 of 30] of *ApoE^−/−^;Senp3^△Mø^* mice compared with 23.33% [7 of 30] of *ApoE^−/−^;Senp3^flox/flox^* mice; *p* < 0.05) (Figure 2I). Notably, the morphological analysis revealed that myeloid‐specific SENP3 deficiency reduced collagen deposition and elastic fiber breakage in abdominal aortic tissues after AngII‐infusion (Figure 2J–L).

Female *ApoE^−/−^;Senp3^△Mø^* mice also exhibited a lower AAA incidence (Figure S7A,B, Supporting Information) and smaller maximal abdominal aortic diameter (Figure S7C, Supporting Information) after AngII infusion, compared to female *ApoE^−/−^;Senp3^flox/flox^* mice. However, no statistically significant differences were found in the incidence of ruptured aneurysms and ratio of aortic weight to body weight between *ApoE^−/−^;Senp3^flox/flox^* and *ApoE^−/−^;Senp3^△Mø^* female mice (Figure S7D,E, Supporting Information). The above results reveal that myeloid‐specific SENP3 deficiency significantly ameliorates AngII‐induced AAA formation.

To further investigate the role of SENP3 in the pathogenesis of AAA, *Senp3^flox/flox^* and *Senp3^△Mø^* mice were subjected to another experimental AAA formation model, involving the periaortic application of CaCl_2_.^[^ ^15^ ^]^ Myeloid‐specific SENP3 deficiency significantly mitigated CaCl_2_‐induced AAA formation and reduced the maximal abdominal aortic diameter in both male and female mice (Figures S8 and S9, Supporting Information). Altogether, these results suggest that myeloid‐specific SENP3 deficiency inhibits AAA formation in both AngII‐ and CaCl_2_‐induced models.

### 2.4 SENP3 Deficiency Represses AAA Lesion Macrophage Infiltration and Inflammatory Response

**Figure 3:**
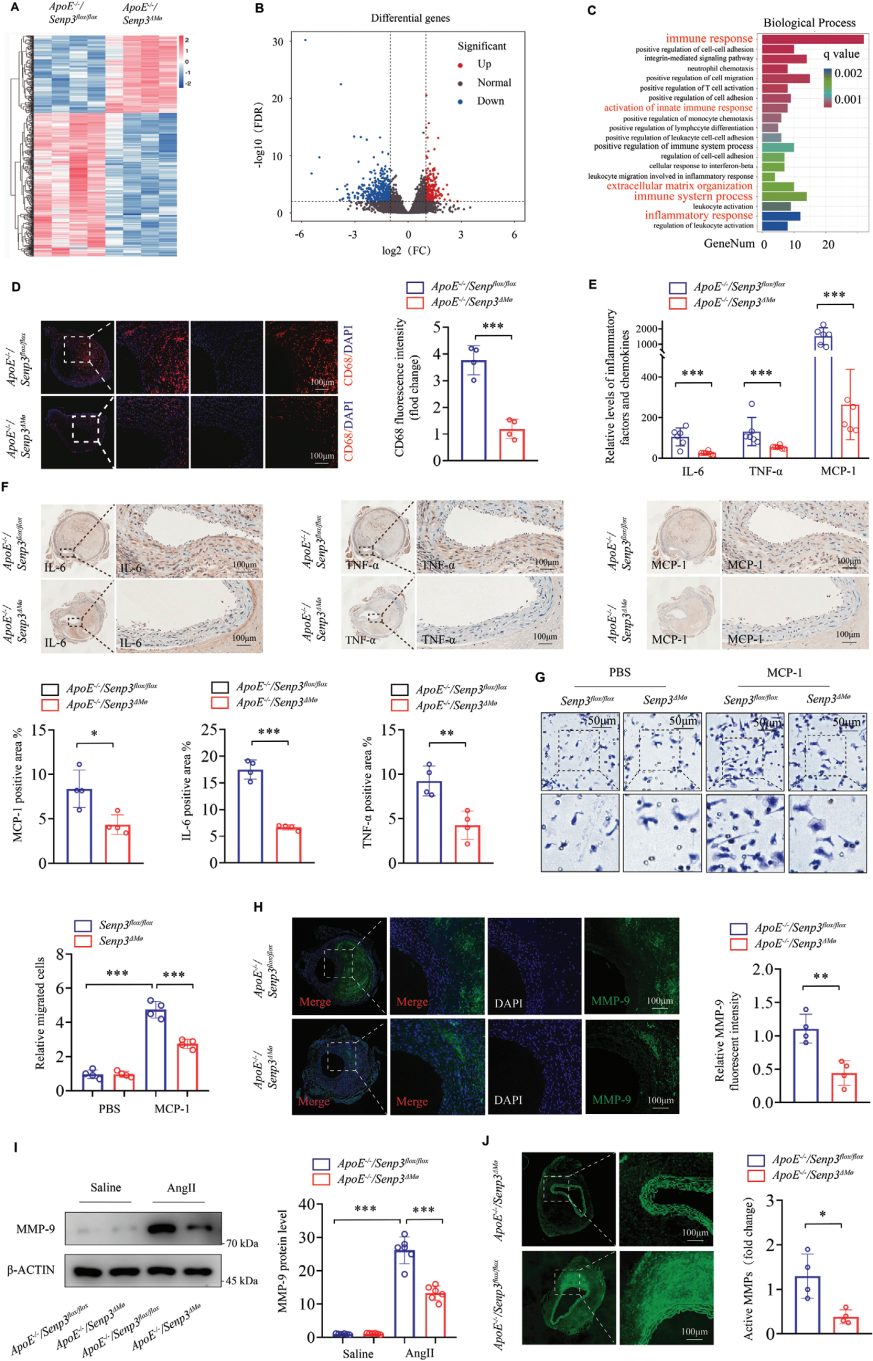
Myeloid‐specific SENP3 deficiency represses AAA Lesion macrophage infiltration and inflammatory response. A) The heatmap shows differentially expressed genes (DEGs) identified by RNA‐seq in aortic tissues from *ApoE^−/−^;Senp3^flox/flox^* and *ApoE^−/−^;Senp3^△Mø^* mice infused with AngII for 28 days. Each column represents an individual replicate, and each row represents an individual gene. Upregulated genes are shown in red, and downregulated genes are displayed in blue. N = 4 for each group. Genes with an adjusted *p*‐value < 0.01 and Fold Change ≥ 2 found by DESeq2 were assigned as differentially expressed. B) Volcano plot reveals the magnitude and significance of DEGs in aortic tissues from AngII‐infused *ApoE^−/−^;Senp3^flox/flox^* and *ApoE^−/−^;Senp3^△Mø^* mice. C) Gene Ontology (GO) pathway enrichment analysis of DEGs in aortic tissues from AngII‐infused *ApoE^−/−^;Senp3^flox/flox^* and *ApoE^−/−^;Senp3^△Mø^* mice. D) Representative images of immunofluorescence staining for CD68 (marker for macrophage) in abdominal aortic samples from *ApoE^−/−^;Senp3^flox/flox^* and *ApoE^−/−^;Senp3^△Mø^* mice infused with AngII for 28 days. Scale bar: 100 µm. Quantification of fluorescence intensity of CD68 (*n*  =  4 for each group). E) Serum levels of IL‐6, TNF‐α, and MCP‐1 in *ApoE^−/−^;Senp3^flox/flox^* and *ApoE^−/−^;Senp3^△Mø^* mice infused with AngII for 28 days were assessed by ELISA. F) Immunohistochemistry analysis of IL‐6, TNF‐α, and MCP‐1 in abdominal aortas from *ApoE^−/−^;Senp3^flox/flox^* and *ApoE^−/−^;Senp3^△Mø^* mice infused with AngII for 28 days. Scale bar: 100 µm. G) Representative images of transwell migration assay and quantification of migrated cells (number/field) in BMDMs isolated from *Senp3^flox/flox^* and *Senp3^△Mø^* mice. Scale bar: 50 µm. H) Representative images of dual immunofluorescence staining of MMP‐9 (green) and CD68 (red) in abdominal aortic samples from *ApoE^−/−^;Senp3^flox/flox^* and *ApoE^−/−^;Senp3^△Mø^* mice infused with AngII for 28 days. Where indicated, nuclei were counterstained with DAPI (blue). Scale bar: 100 µm. I) MMP9 protein levels were determined by western blot in abdominal aortic samples of *ApoE^−/−^;Senp3^flox/flox^* and *ApoE^−/−^;Senp3^△Mø^* mice infused with AngII or saline for 28 days. J) In situ zymography for gelatinase activity in abdominal aortic samples from *ApoE^−/−^;Senp3^flox/flox^* and *ApoE^−/−^;Senp3^△Mø^* mice infused with AngII for 28 days. Scale bar: 100 µm. Data represent mean ± SEM. *p* values were determined using the student's *t*‐test (D‐F,H‐J) and two‐way ANOVA followed by the Bonferroni post hoc test (G). For all panels, *p < 0.05; **p < 0.01; ***p < 0.001.

In order to further interrogate the role of SENP3 in driving inflammatory vascular diseases, we performed high‐throughput RNA sequencing (RNA‐seq) of aortic tissues from *ApoE^−/−^;Senp3^flox/flox^* and *ApoE^−/−^;Senp3^△Mø^* mice infused with AngII for 28 days. We identified significant differences in the expression of genes associated with immunity and inflammation between the two groups (**Figure** **3**A–C). We therefore evaluated whether myeloid‐specific SENP3 deficiency ameliorates vascular inflammation in AAA. Immunofluorescence staining for CD68 revealed a significant reduction in macrophage infiltration in AAA tissues from *ApoE^−/−^;Senp3^△Mø^* mice compared to those from *ApoE^−/−^;Senp3^flox/flox^* mice (Figure 3D). To gain a deeper understanding of the inflammatory response, we assessed the expression of inflammatory factors using ELISA and immunohistochemistry. Our findings indicated that myeloid‐specific SENP3 deficiency significantly reduced the expression of inflammatory mediators (including IL‐6, TNF‐α, and MCP‐1) both in serum and abdominal aortic tissues after AngII infusion (Figure 3E,F). Furthermore, we found that SENP3‐deficient BMDMs exhibited decreased MCP‐1‐induced cell migration compared to control BMDMs (Figure 3G).

Matrix metalloproteinases (MMPs) play a crucial role in the pathological process of AAA, especially VSMC‐derived MMP‐2 and macrophage‐derived MMP‐9.^[^ ^16^ ^]^ We therefore, examined the expression and activity of MMP‐9 in aortic tissues from *ApoE^−/−^;Senp3^flox/flox^* and *ApoE^−/−^;Senp3^△Mø^* mice infused with AngII or saline. Immunofluorescence staining and western blot analysis showed that 28 days of AngII‐infusion significantly upregulated the expressions of MMP‐9 in aortic tissues from *ApoE^−/−^;Senp3^flox/flox^* mice (Figure 3H,I). Additionally, MMP activity was assessed in vivo by an in situ zymography assay. As shown in Figure 3J, myeloid‐specific SENP3 deficiency decreased MMP activity in AAA. Altogether, these results suggest that SENP3 deficiency inhibits macrophage infiltration and inflammatory response during AAA formation.

### 2.5 SENP3 Deficiency Mitigates Ferroptosis in Macrophage

**Figure 4:**
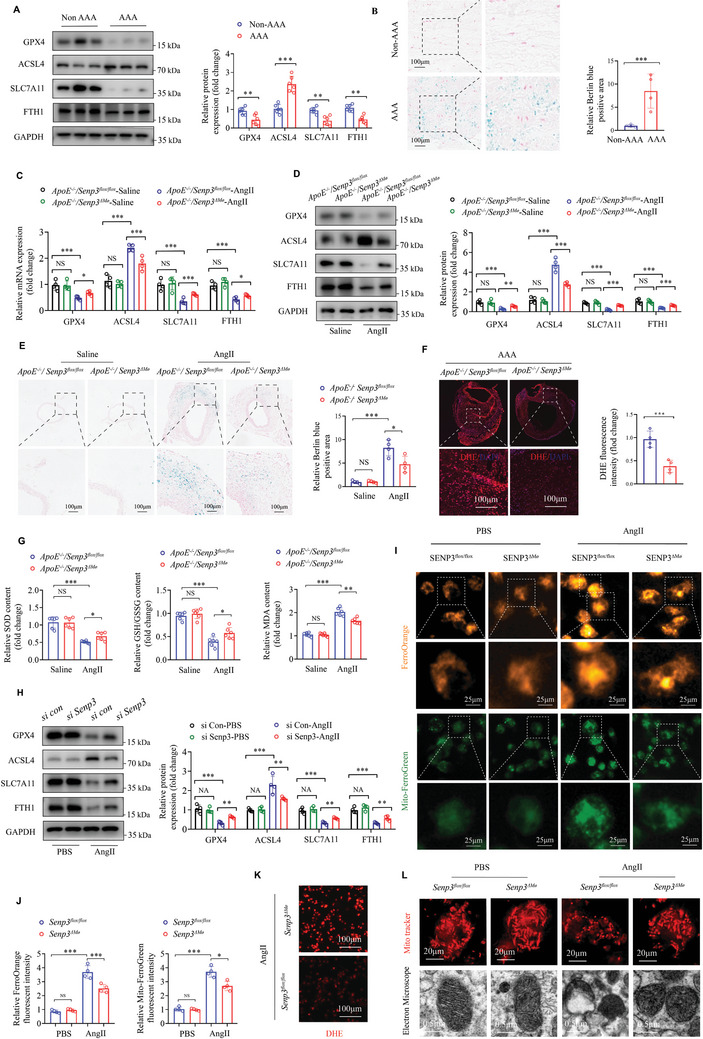
SENP3 deficiency mitigates ferroptosis in macrophage. A) The protein levels of GPX4, ACSL4, SLC7A11, and FTH1 were determined by western blot in human AAA samples and adjacent nonaneurysmal control samples (non‐AAA) (*n* = 6 per group). B) Representative images and quantitative analysis of Berlin blue staining of iron in human non‐AAA and AAA samples (scale bar, 100 µm, *n* = 4). C,D) The mRNA levels and protein levels of GPX4, ACSL4, SLC7A11, and FTH1 were determined by RT‐qPCR (C) and western blot (D) in abdominal aortic samples from *ApoE^−/−^;Senp3^flox/flox^* and *ApoE^−/−^;Senp3^△Mø^* mice infused with AngII or saline for 28 days. E) Representative images and quantitative analysis of berlin blue staining of iron in abdominal aortic samples from *ApoE^−/−^;Senp3^flox/flox^* and *ApoE^−/−^;Senp3^△Mø^* mice infused with AngII or saline for 28 days (scale bar, 100 µm, *n* = 4). F) Representative images and quantitative analysis of dihydroethidium (DHE) staining in abdominal aortic samples from *ApoE^−/−^;Senp3^flox/flox^* and *ApoE^−/−^;Senp3^△Mø^* mice infused with AngII for 28 days (scale bar, 100 µm, *n* = 4). G) The ratio of reduced glutathione (GSH) to oxidized glutathione (GSSG) (GSH/GSSG) and levels of superoxide dismutase (SOD) and malondialdehyde (MDA) in aortic tissues from *ApoE^−/−^;Senp3^flox/flox^* and *ApoE^−/−^;Senp3^△Mø^* mice infused with AngII or saline for 28 days were measured. H) THP‐1 were transfected with control siRNA (si‐con) and Senp3 siRNA (si‐Senp3) for 48 h. The protein levels of GPX4, ACSL4, SLC7A11, and FTH1 were determined by western blot. I,J) The levels of cellular ferrous iron (Fe^2+^) and mitochondrial Fe^2+^ were assessed by FerroOrange probes and Mito‐FerroGreen probes in BMDMs which were isolated from *Senp3^flox/flox^* and *Senp3^△Mø^* mice and stimulated with AngII (1 µmol L^−1^) or PBS for 24 h (scale bar, 100 µm, *n* = 4). K) Representative images of DHE staining in BMDMs isolated from *Senp3^flox/flox^* and *Senp3^△Mø^* mice and stimulated with AngII (1 µmol L^−1^) for 24 h (scale bar, 100 µm, *n* = 4). L) Representative images of Mito tracker (above) and electron micrograph of mitochondria (below) in BMDMs isolated from *Senp3^flox/flox^* and *Senp3^△Mø^* mice and stimulated with AngII (1 µmol L^−1^) or PBS for 24 h (scale bar, 20 or 0.5 µm, *n* = 4). Data represent mean ± SEM. *p* values were determined using the student's *t*‐test (A, B, F) and two‐way ANOVA followed by Bonferroni post‐hoc test (C–E,G–J). For all panels, *p < 0.05; **p < 0.01; ***p < 0.001; ns: not significant (*p* > 0.05).

Numerous studies have shown that macrophage ferroptosis is strongly implicated in aneurysmal and other inflammatory diseases.^[^ ^8^ ^.^ ^17^, ^18^ ^]^ To gain additional insight into the potential mechanisms underlying how SENP3 regulates macrophage inflammation and AAA development, we speculate the potential role of SENP3 in macrophage ferroptosis in the pathogenesis of AAA. Our findings demonstrated the activation of ferroptosis in human aneurysms, as indicated by decreased protein levels of ferroptosis‐related proteins including GPX4, SLC7A11, and FTH1, and increased ACSL4 proteins (**Figure** **4**A). In addition, we found excess iron deposition within the AAA lesions of human samples (Figure 4B), consistent with previous findings.^[^ ^7^, ^19^ ^]^ We therefore conducted a preliminary investigation into the impact of SENP3 on ferroptosis. The impact of myeloid‐specific SENP3 deficiency on ferroptosis in AngII‐induced AAA mouse model was investigated. Using RT‐qPCR on AAA specimens from *ApoE^−/−^;Senp3^flox/flox^* and *ApoE^−/−^;Senp3^△Mø^* mice, we found significant changes in ferroptosis indicators (Figure 4C). Western blot analysis showed no significant differences in protein levels of ferroptosis‐related proteins in aortic tissues between *ApoE^−/−^;Senp3^flox/flox^* and *ApoE^−/−^;Senp3^△Mø^* mice at baseline. However, the aortic tissues from *ApoE^−/−^;Senp3^△Mø^* mice exhibited partial restoration of aberrant protein levels of GPX4, ACSL4, SLC7A11, and FTH1 after 28 days of AngII infusion, compared with those in *ApoE^−/−^;Senp3^flox/flox^* mice (Figure 4D). Moreover, there was less iron deposition in aortic tissues from *ApoE^−/−^;Senp3^△Mø^* mice than in aortic tissues from *ApoE^−/−^;Senp3^flox/flox^* mice treated with AngII (Figure 4E), accompanied by significantly decreased ROS levels measured by DHE intensity (Figure 4F). In addition, the ratio of reduced glutathione (GSH) to oxidized glutathione (GSSG) (GSH/GSSG) and levels of superoxide dismutase (SOD) and malondialdehyde (MDA) in aortic tissues from *ApoE^−/−^;Senp3^flox/flox^* and *ApoE^−/−^;Senp3^△Mø^* mice infused with AngII or saline were further measured. The results showed that GSH/GSSG and SOD levels were both decreased, whereas MDA levels were increased in aortic tissues of *ApoE^−/−^;Senp3^flox/flox^* mice after AngII infusion (Figure 4G). Notably, myeloid‐specific SENP3 deficiency significantly inhibited AngII‐induced decreases in GSH/GSSG and SOD levels, as well as increases in MDA production in aortic tissues (Figure 4G). The above results suggest that SENP3 deficiency mitigates ferroptosis in AAA.

To further verify the role of SENP3 in AngII‐induced ferroptosis, primary BMDMs were isolated from *Senp3^flox/flox^* and *Senp3^△Mø^* mice and were stimulated with AngII (1 µmol L^−1^) for 24 h. Consistent with in vivo data, we found that SENP3‐deficient BMDMs exhibited increased GPX4 and SLC7A11 proteins after AngII stimulation (Figure S10A–D, Supporting Information), which was further demonstrated in human THP‐1 cells (Figure 4H). In contrast, SENP3 overexpression exacerbated the changes of ferroptosis markers such as GPX4, ACSL4, SLC7A11, and FTH1 in BMDMs after AngII stimulation (Figure S11, Supporting Information). In addition, the levels of cellular Fe^2+^ and mitochondrial Fe^2+^ were further assessed by FerroOrange and Mito‐FerroGreen probes in WT and SENP3‐deficient BMDMs. Our findings indicated that following AngII stimulation, the elevation of Fe^2+^ levels was significantly less pronounced in BMDMs isolated from *Senp3^△Mø^* mice, compared to those from *Senp3^flox/flox^* mice (Figure 4I,J). Moreover, SENP3 deficiency reversed the adverse events induced by AngII treatment, including increased cell death, ferrous iron (Fe^2+^) level, ROS generation, and MDA production, as well as decreased SOD and GSH/GSSG levels in BMDMs in vitro (Figure S10E–J, Supporting Information; Figure 4K), suggesting the protective effect of SENP3 deficiency on AngII‐induced ferroptosis in macrophages.

It has been well demonstrated that the progression of ferroptosis predominantly induces mitochondrial morphological changes, including decrease in mitochondrial volume, increased mitochondrial membrane density, decreased or disappeared mitochondrial cristae, and rupture of the mitochondrial outer membrane.^[^ ^20^, ^21^ ^]^ We therefore investigated the impact of myeloid‐specific SENP3 deficiency on mitochondrial morphology. Utilizing confocal microscopy and transmission electron microscopy, we found that SENP3 deficiency partially restored mitochondrial ultrastructure damage in BMDMs after AngII stimulation (Figure 4L).

Taken together, these data suggest that SENP3 deficiency mitigates ferroptosis in macrophage in vitro and in AAA in vivo.

### 2.6 SENP3 Regulates AAA by Targeting CTH to Regulate Ferroptosis Signals

**Figure 5:**
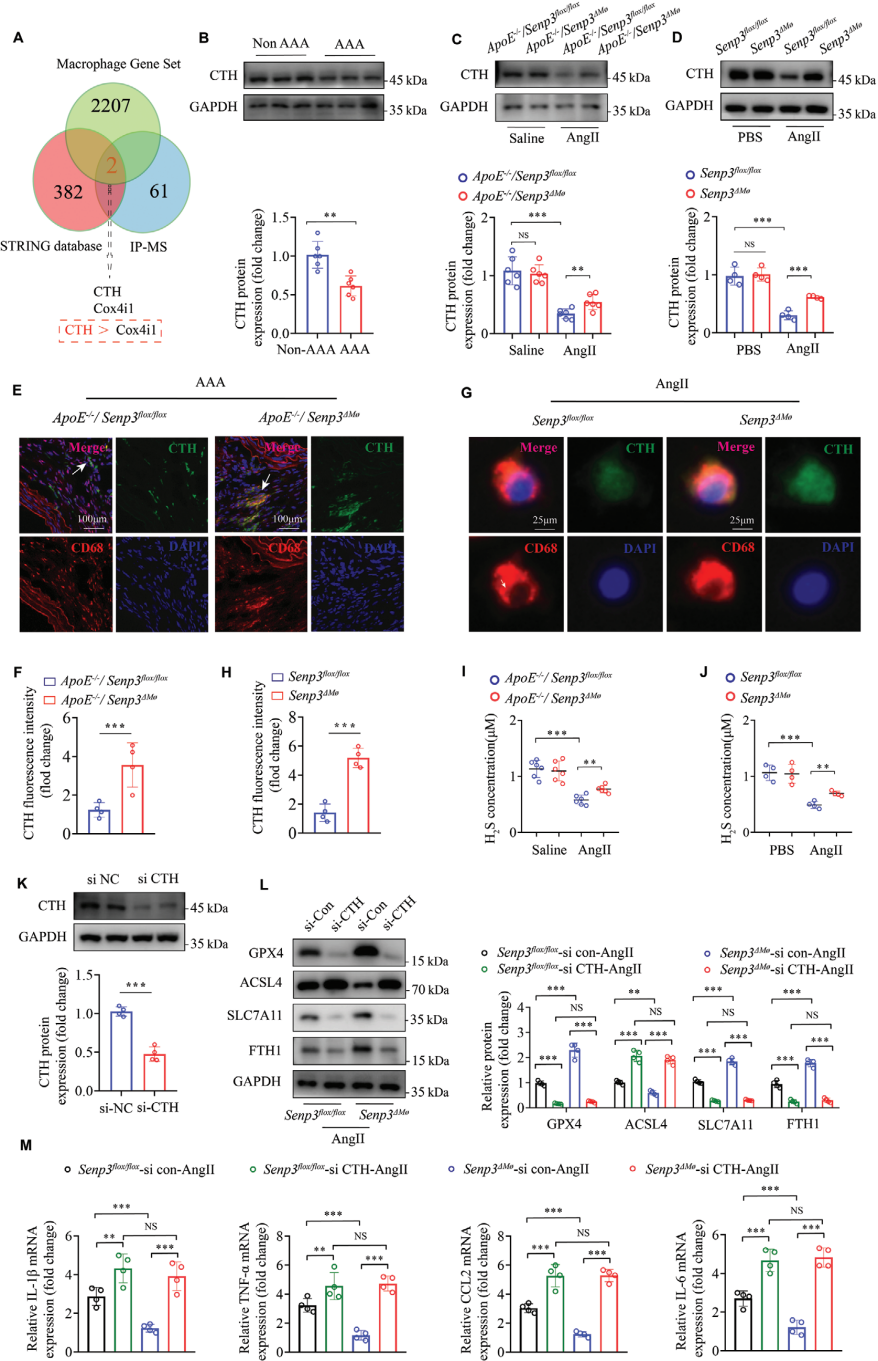
SENP3 regulates AAA by targeting CTH to regulate ferroptosis signals. A) By utilizing IP‐MS in conjunction with the STRING database based on PI3K/AKT signaling pathways, as well as macrophage gene set, two overlapping targets, CTH and Cox4i1, were identified. In the IP‐MS analysis results, the enrichment score of CTH was higher than that of Cox4i1. B) CTH protein levels were determined by western blot in human AAA samples and adjacent nonaneurysmal control samples (non‐AAA) (*n* = 6 per group). C) CTH protein levels were determined by western blot in abdominal aortic samples of *ApoE^−/−^;Senp3^flox/flox^* and *ApoE^−/−^;Senp3^△Mø^* mice infused with AngII or saline for 28 days. D) CTH protein levels were determined by western blot in BMDMs which were isolated from *Senp3^flox/flox^* and *Senp3^△Mø^* mice and stimulated with AngII (1 µmol/L) for 180 min. E,F) Representative images of dual immunofluorescence staining for CTH (green) and CD68 (red) in abdominal aortic samples of *ApoE^−/−^;Senp3^flox/flox^* and *ApoE^−/−^;Senp3^△Mø^* mice infused with AngII for 28 days. Where indicated, nuclei were counterstained with DAPI (blue). G,H) Representative images of dual immunofluorescence staining for CTH (green) and CD68 (red) in BMDMs which were isolated from *Senp3^flox/flox^* and *Senp3^△Mø^* mice and stimulated with AngII (1 µmol L^−1^) for 180 min. Where indicated, nuclei were counterstained with DAPI (blue). I) The plasma levels of H_2_S in *ApoE^−/−^;Senp3^flox/flox^* and *ApoE^−/−^;Senp3^△Mø^* mice infused with saline or AngII for 28 days were determined (*n* = 6 for each group). J) H_2_S concentrations were determined for the cellular homogenate of BMDMs which were isolated from *Senp3^flox/flox^* and *Senp3^△Mø^* mice and stimulated with AngII (1 µmol L^−1^) for 24 h. K) BMDMs were transfected with control siRNA (si‐NC) and CTH siRNA (si‐CTH) for 48 h. The knockout efficiency of CTH was verified by western blot. L) The protein levels of GPX4, ACSL4, SLC7A11, and FTH1 were determined by western blot in BMDMs which were isolated from *Senp3^flox/flox^* and *Senp3^△Mø^* mice and were further transfected with si‐NC and si‐CTH for 48 h before stimulation with AngII (1 µmol L^−1^) for 24 h. M) BMDMs isolated from *Senp3^flox/flox^* and *Senp3^△Mø^* mice were transfected with si‐NC and si‐CTH for 48 h before stimulation with AngII (1 µmol L^−1^) for 24 h. The mRNA levels of IL‐1β, TNF‐α, CCL2, and IL‐6 were determined by RT‐qPCR. *p* values were determined using student's t‐test (B, F, H, and K) and two‐way ANOVA followed by Bonferroni post‐hoc test (C,D,I,J,L,M). For all panels, *p < 0.05; **p < 0.01; ***p < 0.001; ns: not significant (*p* > 0.05).

To clarify the mechanism by which SENP3 regulates AAA development, we reviewed the RNA‐seq data for aortic tissues from AngII‐infused *ApoE^−/−^;Senp3^flox/flox^* and *ApoE^−/−^;Senp3^△Mø^* mice. Bioinformatics analyses of differentially expressed genes suggested that PI3K/Akt signaling pathway was one of the most remarkable pathways enriched (data not shown). By utilizing IP‐MS quantitative data in conjunction with the STRING database based on the PI3K/Akt signaling pathways, as well as the macrophage gene set, 2 overlapping targets were identified, with CTH standing out as a crucial enzyme in the production of vascular H_2_S and a pivotal regulator of vascular function (**Figure** **5**A). Additionally, the established link between CTH and diverse biological processes such as macrophage inflammation, ferroptosis, and aneurysm development underscores its significance.^[^ ^22^, ^23^, ^24^, ^25^ ^]^ Therefore, we examined whether SENP3 acts through CTH to modulate the AAA pathology. Our study revealed a reduction in the protein expression of CTH in human aneurysm tissues relative to non‐aneurysmal tissues (Figure 5B), aligning with existing literature.^[^ ^26^ ^]^ Consistently, CTH protein level was significantly decreased in aortic tissues from *ApoE^−/−^;Senp3^flox/flox^* after AngII infusion (Figure 5C). However, our results indicated that the downregulation of CTH in aortic tissues was partially reversed in *ApoE^−/−^;Senp3^△Mø^* mice after AngII infusion (Figure 5C). Additionally, our findings demonstrated that following AngII stimulation, the reduction in CTH expression was significantly restored in BMDMs isolated from *Senp3^△Mø^* mice compared to those isolated from *Senp3^flox/flox^* mice (Figure 5D).

As CTH functions as a pivotal enzyme in the biosynthesis of vascular H_2_S,^[^ ^27^ ^]^ we assessed H_2_S concentrations across various experimental groups in both in vivo and in vitro systems. We found that H_2_S levels were dramatically decreased in plasma of *ApoE^−/−^;Senp3^flox/flox^* mice after AngII infusion (Figure 5I). Myeloid‐specific SENP3 deficiency significantly reversed the decreases in H_2_S levels after AngII infusion (Figure 5I). This was further confirmed in primary BMDMs in vitro (Figure 5J). Collectively, the above results suggest that SENP3 negatively regulates CTH expression and H_2_S production.

To investigate the potential involvement of CTH in mediating the effects of SENP3 deficiency on ferroptosis, we knocked down CTH expression by transfecting BMDMs with siRNA targeting CTH (Figure 5K). We observed that CTH knockdown significantly abolished SENP3 deficiency‐induced increases in ferroptosis‐related proteins GPX4, SLC7A11, and FTH1 and decreases in ACSL4 proteins in BMDMs after AngII stimulation (Figure 5L). Consistently, we found that CTH knockdown blocked the effect of SENP3 deficiency on AngII‐induced inflammatory cytokine production in BMDMs (Figure 5M). Moreover, we further demonstrated that pharmacological inhibition of CTH with DL‐Propargylglycine (PAG) also attenuated the protective effect of SENP3 deficiency on AngII‐induced ferroptosis. Specifically, SENP3 deficiency decreased cell death, Fe^2+^ level, ROS generation, and MDA production, as well as increased SOD and GSH/GSSG levels in BMDMs after AngII stimulation. Nevertheless, the protective effects were attenuated in the presence of PAG (Figure S12, Supporting Information). These findings suggest that SENP3 deficiency may modulate ferroptosis through its regulation of CTH expression.

### 2.7 SENP3 De‐SUMOylates CTH and Regulates its Protein Stability

**Figure 6:**
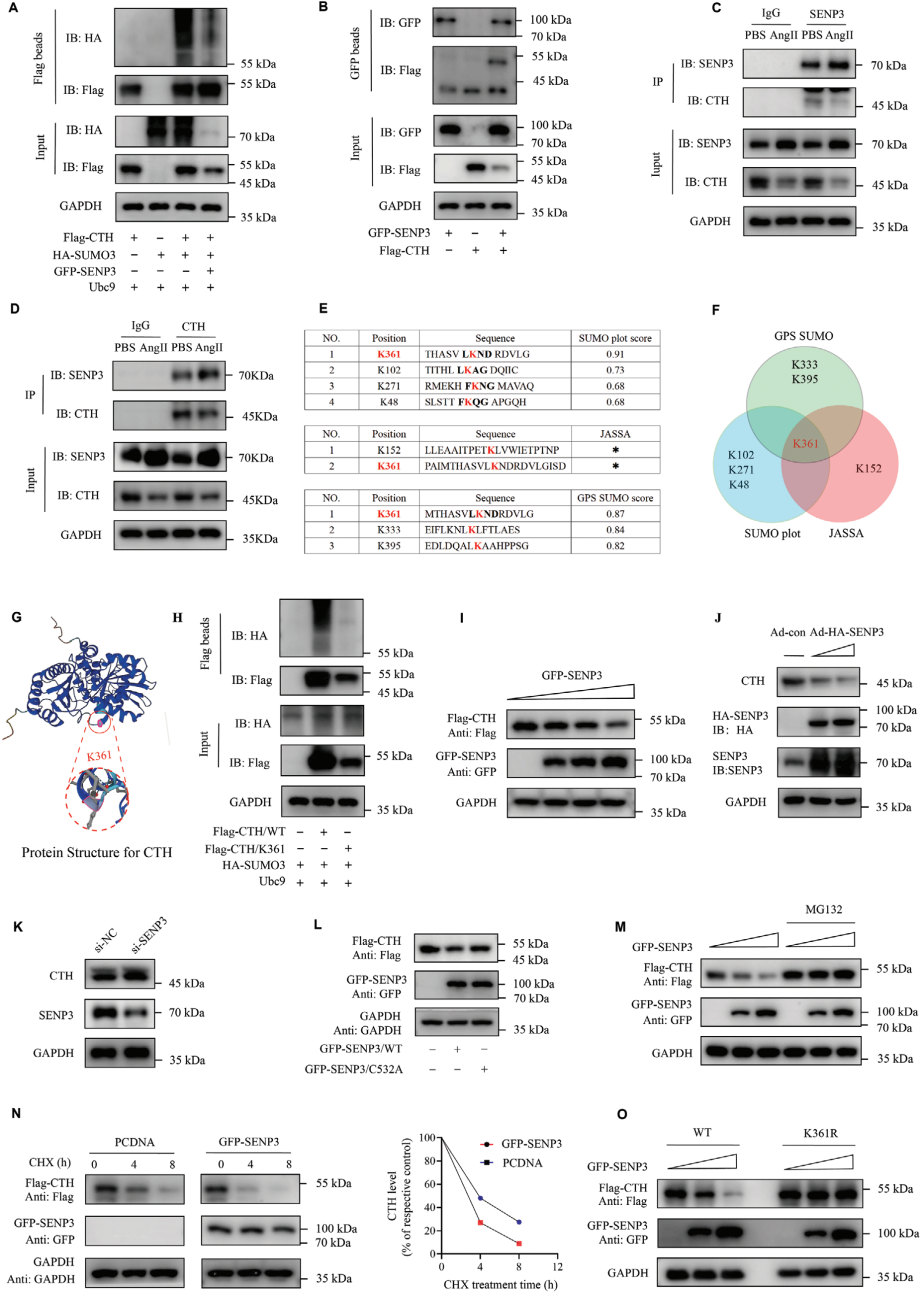
SENP3 de‐SUMOylates CTH and regulates its protein stability. A) 293T cells were transfected with Flag‐CTH, HA‐SUMO3, GFP‐SENP3, and Ubc‐9 for 48 h. The SUMOylation of Flag‐CTH was determined by the immunoprecipitation (IP) assay using Flag beads and western blot using anti‐Flag, anti‐HA, and anti‐GAPDH antibodies. B) Exogenous interaction between SENP3 and CTH was determined by co‐immunoprecipitation (co‐IP) using GFP‐beads. C,D) The endogenous interaction between SENP3 and CTH was determined in BMDMs stimulated with AngII by coimmunoprecipitation assay using antibody against SENP3 or CTH. E,F) SUMOplotTM Analysis Program online tool (https://www.abcepta.com/sumoplot, accessed on 11 February 2023), JASSA, and GPS‐SUMO were employed to predict the SUMOylation sites of CTH. G) Structure of CTH protein and site of K361. H) 293T cells were transfected with Flag‐CTH/WT or Flag‐CTH/K361R, HA‐SUMO3, and Ubc‐9 for 48 h. The SUMOylation of Flag‐CTH was determined by the IP assay using Flag beads and western blot using anti‐Flag, anti‐HA, and anti‐GAPDH antibodies. I) 293T cells were transfected with Flag‐CTH and increasing amounts of GFP‐SENP3 for 48 h. The protein levels of Flag‐CTH, GFP‐SENP3, and GAPDH in whole cell lysates were determined by western blot with anti‐Flag, anti‐GFP, and anti‐GAPDH antibodies. J) BMDMs were infected with increasing amounts of adenovirus expressing HA‐SENP3 for 48 h. The levels of CTH and HA‐SENP3 in whole cell lysates were determined by western blot with anti‐CTH, anti‐HA, anti‐SENP3, and anti‐GAPDH antibodies. K) 293T cells were transfected with control siRNA (si‐NC) and SENP3 siRNA (si‐SENP3) for 48 h. Lysates were prepared and analyzed by western blot. L) 293T cells were transfected with Flag‐CTH and equal amounts of PCDNA or GFP‐SENP3 or GFP‐SENP3/C532A for 48 h. Lysates were prepared and analyzed by western blot. M) 293T cells were transfected with Flag‐CTH and increasing amounts of GFP‐SENP3 for 48 h, in the presence or absence of MG132 (10 µmol L^−1^) for the last 10 h. Lysates were prepared and analyzed by western blot. N) 293T cells were transfected with Flag‐CTH and PCDNA or GFP‐SENP3 for 36 h and were subsequently exposed to the protein synthesis inhibitor cycloheximide (CHX) for the indicated time. Lysates were prepared and analyzed by western blot. The relative level of CTH was evaluated by densitometry and normalized to GAPDH. O) 293T cells were transfected with Flag‐CTH/WT or Flag‐CTH/K361R and increasing amounts of GFP‐SENP3 for 48 h. The levels of Flag‐CTH in whole‐cell lysates were determined by western blot with anti‐Flag, anti‐GFP, and anti‐GAPDH antibodies.

Given that SENP3 is a SUMO2/3‐specific protease, it is hypothesized that CTH may undergo SUMOylation, while SENP3 may catalyze the deSUMOylation of CTH. As shown in **Figure** **6**A, CTH SUMOylation was detected by immunoprecipitation in HEK‐293T cells co‐expressed with Flag‐CTH and HA‐SUMO3, while SENP3 deconjugated SUMO‐3 from CTH. The interaction between CTH and SENP3 was further confirmed by co‐immunoprecipitation assay in exogenous setting and endogenous system of BMDMs (Figure 6B–D). These findings imply that SENP3 de‐SUMOylates CTH via a direct interaction.

To investigate the amino acid residues targeted for SUMOylation on CTH, we employed various computational‐system‐based software including SUMOplotTM, JASSA, and GPS‐SUMO to predict potential SUMOylation sites. As shown in Figure 6E–G, the analysis of SUMOylation sites predicted by the three software sets revealed that only the K361 site exhibited overlap. We therefore mutated the predicted SUMOylation site K361 to arginine (R), constructing CTH site‐specific single‐point mutant CTH‐K361R. The Flag beads pull‐down assay revealed that the SUMOylation of CTH was partially abolished by the K361R single‐point mutation, indicating that K361 is the main SUMOylation site in CTH (Figure 6H). Collectively, our results suggest that CTH is SUMOylated at K361.

It has been well demonstrated that SUMOylation can regulate protein stability of substrate proteins.^[^ ^11^, ^12^, ^28^ ^]^ We then investigated whether SENP3 and SUMOylation regulate CTH protein stability. We found that the protein level of CTH was dose‐dependently decreased by SENP3 overexpression (Figure 6I,J). Knockdown of SENP3 in 293T cells led to upregulation of CTH expression (Figure 6K). As cysteine 532 of SENP3 dominates its enzymatic activity, we overexpressed SENP3‐C532A, an enzymally inactive mutant, and observed that the protein level of CTH was decreased by SENP3‐WT, but not SENP3‐C532A (Figure 6L). This result reveals that the enzymatic activity of SENP3 is essential for the modulation of CTH protein levels. Moreover, we found that SENP3‐mediated CTH elimination was abolished following treatment with the 26S proteasome inhibitor MG132 (Figure 6M). To further demonstrate this point of view, protein synthesis inhibitor cycloheximide (CHX) was used to suppress protein biosynthesis. The half‐life of exogenous CTH was markedly shortened in cells overexpressing SENP3 (Figure 6N). Consistent with these results, SENP3‐mediated CTH downregulation was abolished in the CTH‐K361R mutant form (Figure 6O). Collectively, these findings suggest that SENP3 facilitates the degradation of CTH through a proteasome‐dependent mechanism.

As SENP3 acts as a SUMO2/3‐specific protease, we then examined the effect of SUMOylation (SUMO‐3) on CTH expression. We found that the protein level of CTH was dose‐dependently increased by SUMO‐3 overexpression, which was abolished following treatment with the MG132 (Figure S13A,B, Supporting Information). In addition, the elevated protein level of CTH after SUMO‐3 overexpression was compromised by CTH/K361R mutant (Figure S13C, Supporting Information). To further explore the role of SUMOylation in modulating CTH stability, cells were treated with the CHX. Co‐expression of SUMO‐3 with CTH increased the half‐life of CTH (Figure S13D, Supporting Information). By co‐transfecting CTH, SUMO3, and SENP3, we further confirmed that SUMO‐3 promoted CTH protein stability, whereas SENP3 facilitated its degradation (Figure S13E, Supporting Information). Altogether, these results suggest that SENP3 de‐SUMOylates CTH and regulates its protein stability.

### 2.8 CTH Inhibitor Counteracts the Protective Effect of SENP3 Deficiency on AAA

**Figure 7:**
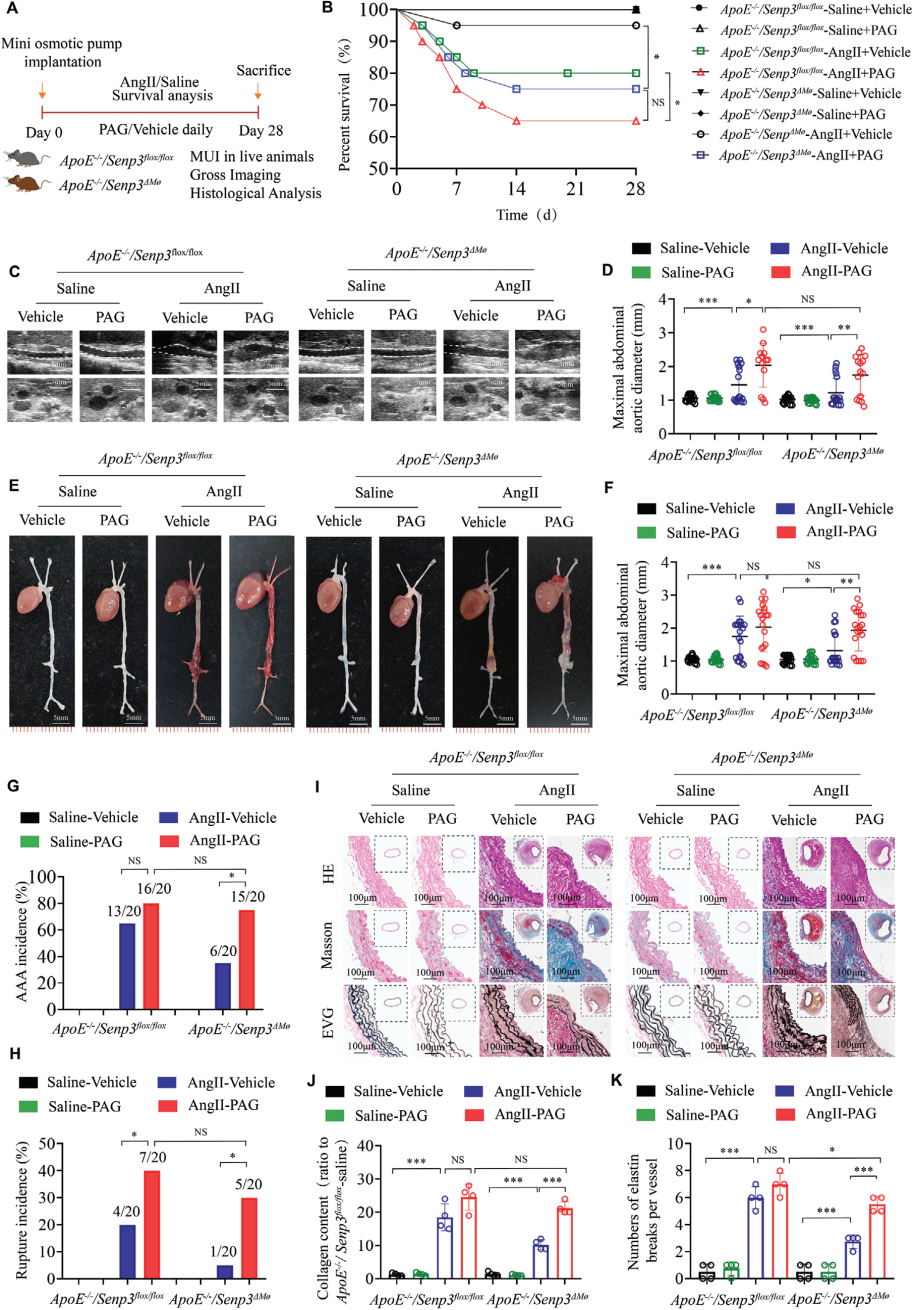
CTH inhibitor counteracts the protective effect of SENP3 deficiency on AAA. A) Schematic protocol: *ApoE^−/−^;Senp3^flox/flox^* and *ApoE^−/−^;Senp3^△Mø^* mice were infused with saline or AngII (1.44 mg kg^−1^ d^−1^) and intraperitoneally injected with DL‐Propargylglycine (PAG) (50 mg kg^−1^ d^−1^) for 28 days (*n* = 20 per group). B) Survival curves in the indicated groups (*n* = 20 per group). Survival data were analyzed by the Kaplan–Meier method and compared using the log‐rank test. C,D) Representative images and quantification of the maximal abdominal aortic diameter monitored by micro‐ultrasound imaging in each group. Scale bar: 5 mm. E) Representative images of the abdominal aorta visualized by macroscopic examination in the indicated groups. Scale bar: 5 mm. F) Quantification of the maximal abdominal aortic diameter measured by a digital vernier caliper for each group. G,H) The incidences of AngII‐induced AAA (G) and aortic rupture (H) in the indicated groups. Data were analyzed by a Fisher exact test. I) Representative images of suprarenal aortic sections stained with hematoxylin and eosin (H&E), Masson Trichrome, and Van Gieson in each group. Scale bar: 100 µm. J,K) Quantification of relative collagen content (J) and numbers of elastin breaks per vessel (K) in each group. Data represents mean ± SEM. *p* values were determined using two‐way ANOVA followed by Bonferroni post‐hoc test (D,F,J,K) and Fisher exact test (G,H). For all panels, *p < 0.05; **p < 0.01; ***p < 0.001; ns: not significant (*p* > 0.05).

To further explore the contribution of CTH signaling to the function of SENP3 in AAA pathology, saline‐ or AngII‐infused *ApoE^−/−^;Senp3^flox/flox^* and *ApoE^−/−^;Senp3^△Mø^* mice were treated with PAG, a selective inhibitor of CTH, at a dose of 50 mg kg^−1^ day^−1^ for 28 days (**Figure** **7**A). As illustrated in Figure 7B, PAG treatment exacerbated the mortality of *ApoE^−/−^;Senp3^flox/flox^* and *ApoE^−/−^;Senp3^△Mø^* mice after AngII‐infusion (20% versus 35% for *ApoE^−/−^;Senp3^flox/flox^* mice; *p* < 0.05; 5% versus 25% for *ApoE^−/−^;Senp3^△Mø^* mice; *p* < 0.05). However, there was no significant difference in the survival rates between AngII‐infused *ApoE^−/−^;Senp3^flox/flox^* and *ApoE^−/−^;Senp3^△Mø^* mice that received PAG treatment (Figure 7B) (65% vs 75%; *p* > 0.05). Moreover, we found that PAG treatment exacerbated aortic enlargement (Figure 7C–F) and increased the incidences of AAA and aortic rupture (Figure 7G,H) in *ApoE^−/−^;Senp3^flox/flox^* and *ApoE^−/−^;Senp3^△Mø^* mice after AngII‐infusion. Notably, we found that the reduced aortic enlargement and reduced incidences of AAA and aortic rupture in *ApoE^−/−^;Senp3^△Mø^* mice after AngII‐infusion in comparison with those in *ApoE^−/−^;Senp3^flox/flox^* mice after AngII‐infusion, were effectively reversed by PAG treatment (Figure 7C–H). In Addition, the better aortic structural integrity and less collagen accumulation and elastic fiber breakage in *ApoE^−/−^;Senp3^△Mø^* mice after AngII‐infusion, compared with those in *ApoE^−/−^;Senp3^flox/flox^* mice after AngII‐infusion, were effectively reversed by PAG treatment (Figure 7I–K), suggesting that pharmacological CTH inhibition counteracts the protective effect of SENP3 deficiency on AAA. Taken together, these findings indicate that the protective effect of SENP3 deficiency in AAA depends on CTH signaling.

### 2.9 Supplementation with the H_2_S Donor ATB346 Protects Against AAA Formation

As a crucial inflammatory mediator in macrophages, H_2_S directly or indirectly modulates various pathophysiological process in cardiovascular disorders such as aneurysms, atherosclerosis, and pulmonary hypertension.^[^ ^29^ ^]^ The promise of H_2_S‐based therapeutics is now being demonstrated in clinical trials, with ATB‐346 emerging as the most advanced compound under scrutiny.^[^ ^30^ ^]^ ATB‐346 is a hydrogen sulfide‐releasing non‐steroidal anti‐inflammatory drug derived from naproxen, which exhibits anti‐inflammatory effects in conditions such as gastrointestinal ulcers/hemorrhages, spinal cord injury, postoperative ileus, and periodontitis.^[^ ^31^, ^32^, ^33^, ^34^ ^]^ Thus, we wondered whether the impaired H_2_S cycle in AAAs would be reactivated by supplementing the key H_2_S donor ATB346.

**Figure 8:**
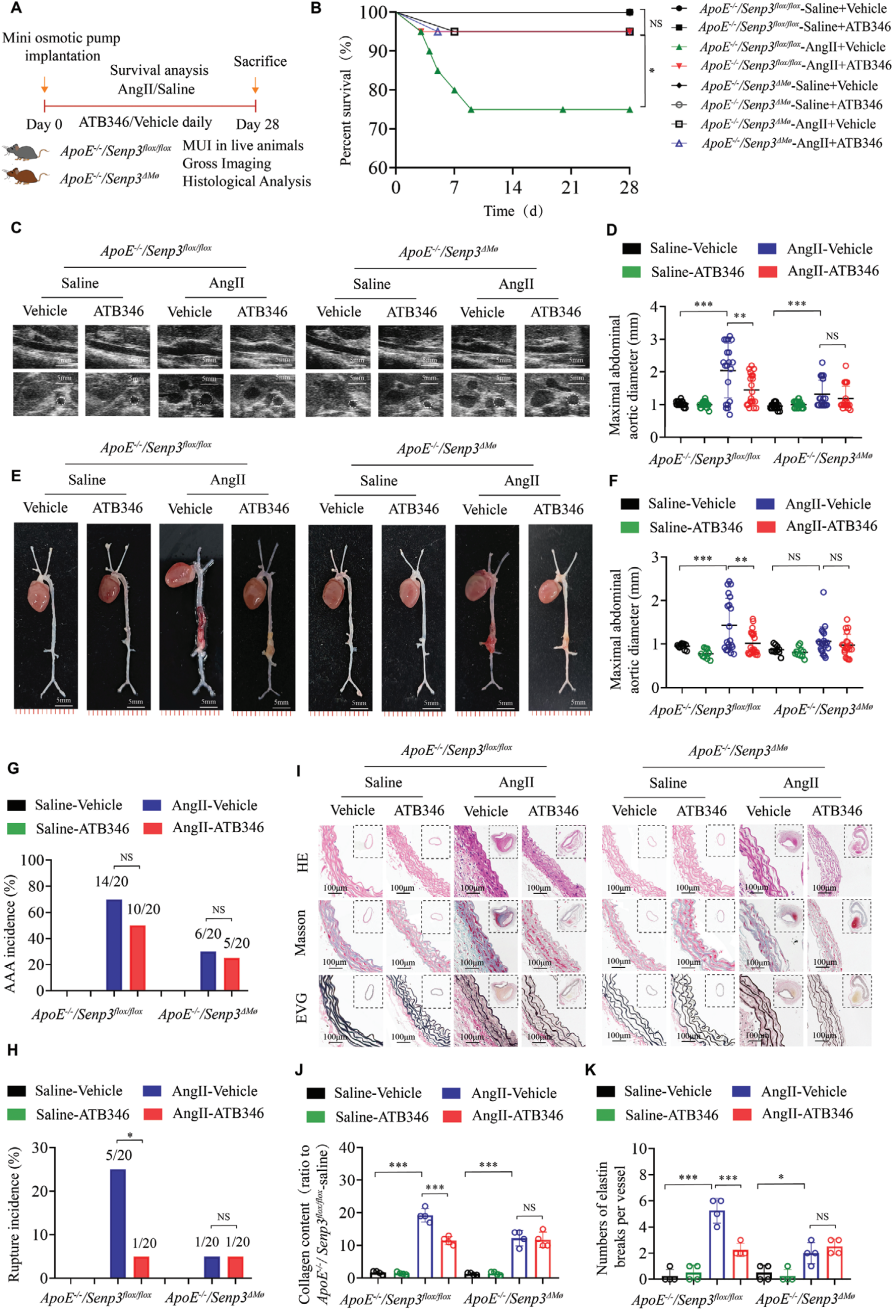
Supplementation with the H_2_S donor ATB346 protects against AAA formation. A) Schematic protocol: *ApoE^−/−^;Senp3^flox/flox^* and *ApoE^−/−^;Senp3^△Mø^* mice were infused with saline or AngII (1.44 mg kg^−1^ d^−1^) and intragastrically injected with ATB346 (16 mg kg^−1^) for 28 days (*n* = 20 per group). B) Survival curves in the indicated groups (*n* = 20 per group). Survival data were analyzed by the Kaplan‐Meier method and compared using the log‐rank test. C,D) Representative images and quantification of the maximal abdominal aortic diameter monitored by micro‐ultrasound imaging in each group. Scale bar: 5 mm. E) Representative images of the abdominal aorta visualized by macroscopic examination in the indicated groups. Scale bar: 5 mm. F) Quantification of the maximal abdominal aortic diameter by a digital vernier caliper for each group. Data were analyzed by two‐way ANOVA followed by the Bonferroni post hoc test. G,H) The incidences of AngII‐induced AAA (G) and aortic rupture (H) in the indicated groups. I) Representative images of suprarenal aortic sections stained with hematoxylin and eosin (H&E), Masson Trichrome, and Van Gieson in each group. Scale bar: 100 µm. J,K) Quantification of relative collagen content (J) and numbers of elastin breaks per vessel (K) in each group. Data represent mean ± SEM. *p* values were determined using two‐way ANOVA followed by Bonferroni post‐hoc test (D,F,J,K) and Fisher exact test (G.H). For all panels, *p < 0.05; **p < 0.01; ***p < 0.001; ns: not significant (*p* > 0.05).

To verify this hypothesis, saline‐ or AngII‐infused *ApoE^−/−^;Senp3^flox/flox^* and *ApoE^−/−^;Senp3^△Mø^* mice were intragastrically administered with ATB346 at a dose of 16 mg kg^−1^ for 28 days. ATB346 supplementation resulted in improved survival in AngII‐infused *ApoE^−/−^;Senp3^flox/flox^* mice (75% vs 95%; *p* < 0.05), but had no significant effects on survival rate in AngII‐infused *ApoE^−/−^;Senp3^△Mø^* mice (95% vs 95%; *p* > 0.05) (**Figure** **8**A,B). Furthermore, our findings indicated that ATB346 improved the maximal internal diameters in AngII‐infused *ApoE^−/−^;Senp3^flox/flox^* mice, while showing no significant effects in AngII‐infused *ApoE^−/−^;Senp3^△Mø^* mice (Figure 8C–F). Although ATB346 supplementation did not significantly decreased AAA incidence in AngII‐infused *ApoE^−/−^;Senp3^flox/flox^* mice (Figure 7G), it significantly decreased the incidence of aortic rupture (Figure 7H) in AngII‐infused *ApoE^−/−^;Senp3^flox/flox^* mice, suggesting that ATB346 supplementation improved AAA pathology in *ApoE^−/−^;Senp3^flox/flox^* mice to some extent. Moreover, ATB346 supplementation significantly reduced pathological collagen accumulation and elastic fiber breakage in abdominal aortic tissues in AngII‐infused *ApoE^−/−^;Senp3^flox/flox^* mice (Figure 8I–K). It is noteworthy that the protective effects of ATB346 on AAA were completely abolished in *ApoE^−/−^;Senp3^△Mø^* mice (Figure 8). In addition, in in vitro system of primary BMDMs, we demonstrated that ATB346 improved AngII‐induced ferroptosis (Figure S14, Supporting Information). Altogether, these results suggest that supplementation with ATB3466 protects against AAA injuries. Moreover, they further confirm our conclusion that SENP3 regulates AAA by targeting CTH to regulate ferroptosis signals.

## 3 Discussion

In this present study, we identified a novel role of the SUMO‐specific protease SENP3 in the pathogenesis of AAA. SENP3 protein expression was upregulated in macrophages in both human and murine AAA specimens. Myeloid‐specific SENP3 deficiency protected mice against AAA formation in both AngII‐ and CaCl_2_‐induced mouse models. Mechanistically, we demonstrated that CTH, a critical enzyme involved in H_2_S production within the vessels, as a novel substrate of SENP3, which mediated the regulatory effects of SENP3 on ferroptosis and inflammatory programs in macrophages. CTH was SUMOylated at Lysine 361 and could be de‐SUMOylated by SENP3. SENP3 facilitated the proteasome‐dependent degradation of CTH. In addition, we further provide evidence demonstrating that pharmacological inhibition of CTH counteracted the protective effect of SENP3 deficiency on AAA formation. Additionally, supplementation with the H_2_S donor ATB346 protected against AAA formation. Overall, the results of the present study provided the first evidence for a novel regulatory SENP3/CTH/ferroptosis signaling axis in AAA formation and progression.

SUMOylation has emerged as an important PTM that regulates protein activity, subcellular localization, stability, and protein‐protein interactions.^[^ ^35^ ^]^ SUMOylation is a dynamic and reversible process, which is catalyzed by SUMO‐specific ligases and reversed by a family of SENPs.^[^ ^10^ ^]^ SENP3, one of the six SENP family members (SENP1–3 and 5–7), has been known as an important regulator of oxidative stress, inflammation, and programmed cell death.^[^ ^36^ ^]^ Recent studies have uncovered several new functions of SENP3 (i.e., regulating inflammatory responses, ribosome biogenesis, and mitochondrial damage) in the pathogenesis of cancer, ischemia‐reperfusion injury, autoimmune and inflammatory diseases.^[^ ^37^, ^38^, ^39^ ^]^ Accumulating evidence has shown that SENP3 regulates cardiovascular physiology/pathology.^[^ ^11^, ^40^, ^41^ ^]^ We have previously demonstrated that SENP3 regulates VSMC function and low‐shear stress‐induced vascular remodeling.^[^ ^11^ ^]^ However, the role of SENP3 in the pathogenesis of AAA has never been investigated. In this present study, we found that SENP3 protein was upregulated in both human and murine AAA specimens in vivo and in BMDMs in vitro after AngII treatment via a ROS‐dependent manner. Myeloid‐specific deletion of SENP3 protected mice against AngII‐ and CaCl_2_‐induced AAA formation. These findings provide compelling evidence of a previously unrecognized role of SENP3 as a critical regulator in the pathogenesis of AAA.

Ferroptosis is an iron‐dependent form of nonapoptotic programmed cell death that is distinct from apoptosis, necrosis, and autophagy^[^ ^42^, ^43^ ^]^ and has been strongly implicated in multiple diseases and pathological conditions, such as degenerative diseases, ischemic disorders, and cardiovascular disease.^[^ ^42^ ^]^ Numerous previous studies have suggested that inhibition of ferroptosis effectively ameliorated the occurrence and development of AAA.^[^ ^7^, ^8^ ^]^ SENP3 has been previously demonstrated to regulate inflammatory, mitochondrial metabolism, and programmed cell death in several organs and cells.^[^ ^11^, ^12^, ^39^ ^]^ Here we provide first evidence demonstrating that SENP3 deficiency mitigated AngII‐induced ferroptosis in macrophage in vitro and in vivo. Moreover, we identified CTH, a critical enzyme involved in H_2_S production, as a novel target protein of SENP3 that mediated the regulatory effects of SENP3 on ferroptosis and inflammatory programs in macrophages in AAA. CTH and the H_2_S signaling pathway have been well‐identified as key regulators of vascular function. For example, CTH deletion exacerbates the progression of AAA and decreased plasma H_2_S levels are associated with an increased risk of AAA.^[^ ^26^ ^]^ Deficiency of CTH promotes aortic elastolysis and medial degeneration in aged mice.^[^ ^44^ ^]^ Inhibition of CTH dampens vasoconstriction in mouse and human intracerebral arterioles.^[^ ^45^ ^]^ Our research indicated that the protective effects of SENP3 deficiency on ferroptosis and AAA pathogenesis were mediated by CTH signaling. We found that CTH was SUMOylated at lysine 361, which could be deconjugated by SENP3. SUMO‐3 promoted CTH protein stability, whereas SENP3 facilitated its proteasome‐dependent degradation. This study identifies the SENP3/CTH axis as a potential novel target for regulating inflammation and ferroptosis in AAA treatment.

In recent years, the role of H_2_S in cardiovascular systems has received increasing attention due to its versatile functions. As a crucial inflammatory mediator in macrophages, H_2_S directly or indirectly modulates various cellular activities, such as phagocytosis, migration, cytokine production, redox homeostasis, and energy metabolism, suggesting it as a promising therapeutic target for inflammatory disease. ATB‐346 is a H_2_S‐donating derivative of the non‐steroidal anti‐inflammatory compound naproxen. Previous preclinical studies have demonstrated that ATB‐346 exhibits anti‐inflammatory effects in conditions such as gastrointestinal ulcers/haemorrhages, spinal cord injury, postoperative ileus, and periodontitis.^[^ ^31^, ^32^, ^33^, ^34^ ^]^ Moreover, the robust anti‐inflammatory properties of ATB‐346 have been validated in human studies of inflammation.^[^ ^46^, ^47^ ^]^ Nevertheless, there are currently no evidence about ATB‐346 being applied in AAA. Based on our experimental findings and given the importance of H_2_S in the physiological and pathological regulation of vasculature, we tentatively applied ATB346 in mouse AAA model. We were therefore surprised to find that supplementation with ATB346 was beneficial to slow down the progression of AAA. However, further investigations to confirm the treatment values are essential for bringing these interventions to the clinic.

In conclusion, we demonstrate that SENP3 accumulates in macrophages of aneurysmal aortic tissues, which promotes macrophage ferroptosis and inflammation via regulation of CTH De‐SUMOylation. The findings provide us with a new perspective for understanding the role of SENP3/CTH/ferroptosis axis in the pathogenesis of AAA. A better understanding of the role of SENP3 and CTH in AAA may ultimately lead to new approaches to prevent and treat aortic aneurysmal diseases.

## 4 Experimental Section

### Human Tissue Specimens

All human aneurysm specimens utilized in this research were obtained from patients who had undergone open abdominal aortic repair, while control samples were extracted from non‐dilated aortic tissue from the same individuals. Patient consent was obtained for the use of tissue samples. The collection and utilization of human specimens adhered to the protocol approved by the Institutional Review Board of Shanghai Jiao Tong University. Participants were recruited with informed consent approved by the Ethics Committee of Shanghai Chest Hospital, Shanghai Jiao Tong University School of Medicine (approval IS21014).

### Animal Experiments

*Senp3^flox/flox^* mice were kindly provided by Prof. Jing Yi from Shanghai Jiao Tong University School of Medicine, Shanghai, China. *Lyz2‐Cre* mice (Cat. NO. NM‐KI‐215037), *ApoE^−/−^* *mice* (Cat. NO. NM‐KO‐190565), and C57BL/6J mice were purchased from Shanghai Model Organisms Center, Inc. *Senp3^flox/flox^* mice were intercrossed with *Lyz2‐Cre* mice to generate *Senp3^flox/flox^*;*Lyz2‐Cre* (*Senp3^△Mø^*) mice, which were further bred with *ApoE^−/−^* mice to establish *ApoE^−/−^;Senp3^flox/flox^*;*Lyz2‐Cre* (*ApoE^−/−^;Senp3^△Mø^*) mice. Male and female mice, aged 8–12 weeks, were utilized in the study. Mice were maintained in controlled conditions of 24 ± 2 °C and 40 ± 5% humidity, subjected to a 12‐h light/dark cycle, and allowed free access to water and food. The animal protocol employed in this research was sanctioned by the Ethics Committee of Shanghai Chest Hospital (Permit Number: KS24062) and adhered to the National Institutes of Health Guidelines for the Care and Use of Laboratory Animals.

### Statistical Analysis

All statistical analyses were performed using GraphPad Prism 6 or SPSS version 20.0. Continuous variables were expressed as mean ± standard error of the mean (SEM), and categorical variables were presented as numbers or percentages. The Shapiro‐Wilk test was utilized to evaluate the normality of the data distribution. For comparisons between two groups, the student's *t*‐test was applied for variables with equal variances, while the unequal variance *t*‐test was used for variables with unequal variances, provided the data were normally distributed. For variables that did not follow a normal distribution, the Mann–Whitney U test was employed. For comparisons involving more than two groups, the Brown‐Forsythe test was initially employed to assess the homogeneity of variances. If the data satisfied the assumption of equal variances, a one‐way ANOVA was conducted, followed by a post hoc analysis using the Bonferroni method to adjust for multiple comparisons. Otherwise, a Welch ANOVA test was performed, followed by a post hoc analysis using the Tamhane T2 method. Two‐way ANOVA followed by Bonferroni post hoc analysis was used when >2 groups and variables were compared. Bivariate comparisons of categorical variables were performed with the χ^2^ test. Survival was estimated by the Kaplan‐Meier method, and differences were evaluated using a stratified log‐rank test. A value of *p* < 0.05 was considered statistically significant.

More details can be found in Supporting Information.

## Conflict of Interest

The authors declare no conflict of interest.

## Author Contributions

L.C., Z.C., D.X., and Y.S. contributed equally to this work. L.C., Z.C., D.X., and Y.S. designed and executed the experiments, and analyzed the data. L.C. wrote the manuscript. Q.X., M.L., Y.J., Y.H., F.L., G.Z., X.W., H.H., and L.F. executed the experiments and provided technical assistance. F.Z. provided technical assistance and acquired the funding. Z.C., Q.S., and B.H. acquired the funding, conceived and designed the study, and revised the manuscript. All authors have read and agreed to the published version of the manuscript.

## Supporting information



Supporting Information

## Data Availability

The data that support the findings of this study are available from the corresponding author upon reasonable request.
