# Supplementary material for: SENP3 Drives Abdominal Aortic Aneurysm Development by Regulating Ferroptosis via De‐SUMOylation of CTH
Source: Adv Sci (Weinh). 2025 Feb 28;12(16):2414500. doi: 10.1002/advs.202414500 (PMC12021093; doi:10.1002/advs.202414500)
Supplement: Supplementary file 1 — Supporting Information [file ADVS-12-2414500-s001.pdf]

ADVANCED SCIENCE
Open Access

Supporting Information

for *Adv. Sci.*, DOI 10.1002/advs.202414500

SENP3 Drives Abdominal Aortic Aneurysm Development by Regulating Ferroptosis via De-SUMOylation of CTH

Long Chen, Zhaohua Cai, Danrui Xiao, Yiping Shi, Qingqing Xiao, Min Liang, Yangjing Jiang, Yijie Huang, Feng Liang, Guo Zhou, Fei Zhuang, Xia Wang, Huanhuan Huo, Liang Fang, Qin Shao* and Ben He**

Supplementary Materials for

SENP3 Drives Abdominal Aortic Aneurysm Development by Regulating Ferroptosis via De-SUMOylation of CTH

Materials and Methods

2.1 Animal experiments

$ApoE^{-/-}$ mice and age-matched Senp3 transgenic mice (*$ApoE^{-/-};Senp3^{flox/flox}$* and *$ApoE^{-/-};Senp3^{\triangle M\o}$* mice) were subcutaneously injected with Angiotensin II (AngII) (Cat# A9525; Sigma-Aldrich, Saint Louis, MO, USA) at a dosage of 1.44 mg/kg/d or saline via osmotic minipumps (model 2004; Alzet, Palo Alto, CA, USA) for 4 weeks as previously described[1]. The superior abdominal aortas, situated between the last pair of intercostal arteries and the right renal branches, were harvested at 28 days post-implantation. An increase in abdominal aortic diameter of 50% or more was indicative of an abdominal aortic aneurysm (AAA). Mice that died during the study were autopsied, and a blood clot outside the dilated aortic wall was considered a ruptured aneurysm.

C57BL/6J mice and age-matched Senp3 transgenic mice (*$Senp3^{flox/flox}$* and *$Senp3^{\triangle M\o}$* mice) were subjected to AAA induction with calcium chloride ($CaCl_2$). After anesthesia, the abdominal aorta between the renal arteries and iliac bifurcation was carefully exposed. Cotton gauze soaked in 0.5 mol/L $CaCl_2$ was attached to the surface of abdominal aorta for 15 minutes. The gauze was then removed, and the intraperitoneal cavity was thoroughly washed with 0.9% sodium chloride (NaCl). Sham control mice received 0.9% NaCl instead of $CaCl_2$. The infrarenal abdominal aorta (located between the right renal branch and iliac artery bifurcation) was harvested at 28 days post-operation.

To further investigate the role of SENP3 in the pathological progression of AAA through the regulation of CTH, we employed DL-Propargylglycine (PAG; Cat# P7888; Sigma-Aldrich, Saint Louis, MO, USA) to suppress CTH in mice. PAG (50 mg/kg/day) treatment was initiated on day 1 after AngII infusion and lasted for 4 weeks. This dose of PAG was safe concerning *in vivo* animal treatment as referenced to similar studies[2, 3]. To investigate the potential reactivation of the impaired H_2S cycle in AAA through supplementation with the key H_2S donor ATB346 (Cat# S6615; Sellect, China), *$ApoE^{-/-};Senp3^{flox/flox}$* and *$ApoE^{-/-};Senp3^{\triangle M\o}$* mice were intragastrically administered with ATB346 (16 mg/kg) from day 1 to 28 after AngII infusion. The dose of ATB346 was deemed safe for in vivo animal treatment, consistent with dosages reported in analogous studies[4-6].

2.2 Tissue collection and processing

Following anesthesia of the mice, serum was obtained by collecting blood from the eyeball, followed by euthanasia via cervical dislocation. Tissue designated for pathological diagnosis or immunofluorescence was fixed in optimal cutting temperature (OCT; Sakura Finetechnic) compound for frozen sections, or in 10% neutral buffer formalin for paraffin embedding. The thickness of paraffin-embedded sections or OCT-embedded sections was 5 μm or 8 μm, respectively. Samples intended for western blot or real-time quantitative polymerase chain reaction (RT-qPCR) analysis were frozen in liquid nitrogen.

2.3 Immunofluorescence and Immunohistochemistry Staining

For immunofluorescence staining, paraffin sections were deparaffinized, rehydrated, and subjected to antigen retrieval (Cat# P0083; Beyotime, China). Frozen sections were fixed in cold methanol at -20°C for 10 minutes. After blocking with 5% normal goat serum for 1 hour, the sections were incubated overnight at 4°C with primary antibodies against SENP3 (1:100; Cat# 5591; Cell Signaling Technology (CST), USA), CD68 (1:50; Cat# ab955; abcam, UK), MMP9 (1:200; Cat# 10375-2-AP; proteintech,

China), GPX4 (1:200; Cat# ab125066; abcam, UK), SLC7A11 (1:200; Cat# ab216876; abcam, UK), CTH (1:200; Cat# 12217-1-AP; proteintech, China), and STUB1 (1:200; Cat# ab134064; abcam, UK). After three washes with PBS, Alexa Fluor 488/555 conjugated secondary antibodies (1:200; Invitrogen, USA) were applied for 1 hour at 37°C in the dark. Finally, the sections were mounted with 4',6-diamidino-2-phenylindole (DAPI) and viewed using fluorescence microscopy.

For immunohistochemistry, paraffin sections were incubated with primary antibodies against IL-6, MCP-1, and TNFα overnight at 4°C after continuous deparaffinization, rehydration, and antigen retrieval process. After washing, sections were incubated in corresponding biotin conjugated secondary antibodies, followed by a 5-minute incubation with 3,3′diaminobenzidine (DAB) peroxidase substrate kit. Representative images were captured by light microscopy

2.4 Protein Extraction and Western Blot

Total proteins were extracted from tissues or cultured cells by RIPA lysis buffer containing PMSF (Beyotime, China). Western blot was performed as briefly mentioned below. Proteins were quantified with a standard BCA assay (Thermo Scientific; Belgium), separated by Sodium dodecyl sulfate-polyacrylamide gel electrophoresis (SDS-PAGE), and transferred to nitrocellulose membranes. Membranes were blocked with 5% non-fat milk dissolved in TBS-T at 37°C for 1 hour, and incubated with primary antibodies at 4°C overnight. After incubation with horseradish peroxidase-conjugated secondary antibodies at 37°C for 1 hour, the membranes were washed 3 times with TBS-T and then visualized with enhanced chemiluminescence (ECL; Millipore). The following antibodies were used: SENP3 (1:1000; Cat# 5591; CST), β-actin (1:5000; Cat# ab6276; Abcam), GAPDH (1:1000; Cat# ab37168; Abcam), GPX4 (1:1000; Cat# ab125066; Abcam), ACSL4 (1:1000; Cat# ab155282; Abcam), SLC7A11 (1:1000; Cat# ab216876; Abcam), FTH1 (1:1000; Cat# ab75973; Abcam), CTH (1:1000; Cat# 12217-1-AP; Proteintech), HA(1:5000; Cat# H9658; Sigma), green fluorescent protein (GFP) (1:1000; Cat# ab290; Abcam), Flag (1:5000; Cat# F1804; Sigma), STUB1 (1:1000; Cat# ab134064; Abcam), MYC (1:1000; Cat# 10828-1-AP; proteintech), MMP9 (1:1000; Cat# ab38898; Abcam).

2.5 RNA Isolation and Real-time Quantitative PCR (RT-qPCR)

Total RNA was extracted from mouse tissues or cultured cells using Trizol reagent (Cat# 15596026; Life technologies, California, USA), according to the manufacturer's instructions. RNA (1 μg) was then reverse-transcribed into first-strand cDNA by primeScript RT reagent kit (Vazyme). Quantitative real-time PCR amplification (RT-qPCR) was conducted using SYBR Premix Ex Taq™ (Takara, Japan) in a LightCycler® 480 Realtime PCR System (Roche Applied Science). The primer sequences used are presented in Table S1. Relative mRNA expression was calculated using the comparative ΔΔCT method and the resulting values were normalized to GAPDH RNA expression.

2.6 Histological Analysis

Tissue paraffin sections were subjected to staining with hematoxylin-eosin (H&E), Masson trichrome, and elastin van Gieson (EVG) to assess general morphology, collagen content, and elastin degradation, respectively. Quantification and grading of elastin degradation involved calculating the number of breaks in each vessel. Collagen content changes in Masson trichrome staining images were analyzed using Image-ProPlus6.0 software.

2.7 Isolation and culture of bone marrow-derived macrophages

Bone marrow-derived macrophages (BMDMs) were collected from the femur and tibia of 6-8-week-old *Senp3*$^{flox/flox}$ and *Senp3*$^{\Delta M\phi}$ mice, as previously described[7]. Briefly, Bone marrow was extracted by flushing the femurs and tibia with PBS using a syringe. The cell suspension was centrifuged at 1000 rpm at 4℃ for 5 minutes, followed by removal of the supernatant and addition of erythrocyte lysate. The mixture was allowed to incubate for 10 minutes before termination of the reaction with medium and subsequent centrifugation for 5 minutes. The resulting cell pellet was resuspended in complete media (RPMI 1640 medium with 10% fetal bovine serum (FBS), 100 U/ mL penicillin, and 100 mg/mL streptomycin) containing 10 ng/mL M-CSF (Cat# 416-ML-050; R&D Systems, Minneapolis, MN, USA) and inoculated into petri dishes. Fresh BMDM growth medium was changed on day 3. Mature BMDMs were ready for further experiments on day 7.

BMDMs were stimulated with the indicated concentrations of AngII for the indicated times. In a separate experiment, BMDMs were pre-treated with PAG (1 mmol/L) for 60 minutes prior to AngII induction[8, 9]. Furthermore, to investigate the impact of ATB346 on ferroptosis, BMDMs were pre-treated with ATB346 (100 μmol/L) for 60 minutes prior to AngII induction[10]. To investigate whether ROS play essential roles in AngII-induced SENP3 upregulation, BMDMs were pre-treated with ROS scavengers, either N-t-butyl-a-phenylnitrone (PBN, 10 mmol/L) or N-acetyl cysteine (NAC, 10 mmol/L) for 60 minutes, and were then subjected to AngII (1 μmol/L) or PBS treatment for another 180 minutes.

2.8 RNA Interference, Plasmid Construction, and Adenovirus Generation

The small interfering RNAs (siRNAs) targeting human SENP3, mouse CTH, mouse STUB1, and human STUB1 were purchased from Genomeditech Co. Ltd. (Shanghai, China) (Table S2). All siRNA transfections were performed using Lipofectamine 3000 Transfection Reagent (Cat# L3000015; Thermo Fisher Scientific, Inc., Waltham, MA, USA), in accordance with the manufacturer's protocol. The knockdown efficiency was confirmed by western blot and RT-qPCR analysis.

Full-length wild-type STUB1 was cloned into the LV011-pHBLV-CMV-MCS-3Flag-EF1-ZsGreen-T2A-Puro vector, whereas full-length wild-type CTH was cloned into the pcDNA3.1-CMV-MCS-3flag-EF1-ZsGreen-T2A-Puro vector (Hanheng Biotechnology (Shanghai) Co., Ltd). The Flag-CTH lysine to argine mutant construct (Flag-CTH/K361R) was generated by site-directed mutagenesis based on the Flag-CTH construct using a QuikChange Mutagenesis Kit (Stratagene, La Jolla, CA). The constructs for GFP-SENP3, GFP-SENP3/C532A, HA-SUMO-3, Myc-ubiquitin (Myc-Ub), and Ubc-9 were used in our previous work[13-15]. The constructs were transiently transfected into cells using Lipofectamine 2000 (Cat# 11668019; Invitrogen, USA) following the manufacturer's instructions.

Adenovirus expressing mouse SNEP3 (Ad-HA-SENP3) and adenovirus expressing empty vector (Ad-con) were purchased from Obio technology (Shanghai, China). For adenovirus infection, primary BMDMs were plated in 6-well plates and infected with Ad-HA-SENP3 and Ad-con at 70% confluence. Growth media were refreshed at 12h and cell were harvested at 48h for further experiments.

2.9 Immunoprecipitation

For immunoprecipitation (IP) assay of exogenously expressed proteins, transfected cells were lysed in cold lysis buffer (Cat# P0013; Beyotime, China) on ice for 30 minutes, followed by centrifugation at 12,000 rpm for 15 minutes. Anti-GFP Magnetic Beads (Cat# P2132; Beyotime, China) or Anti-Flag Magnetic Beads (Cat# P2115; Beyotime, China) was added to cell lysates and incubated at 4°C overnight. The magnetic beads were washed 3 times with TBST. After the last wash, the proteins were eluted with elution buffer and subjected to western blot.

For IP assay of endogenously expressed proteins, cultured cells from different groups were collected and lysed in cold lysis buffer on ice for 30 minutes. The lysates were centrifuged at 12,000 g for 15 minutes at 4°C. The collected supernatants were transferred to a new Eppendorf tube and incubated with primary antibodies or rabbit IgG (Cat# P2267; Beyotime, China) at 4°C overnight on an orbital rotator. After overnight incubation, the antibody/antigen mixtures were incubated with Protein A/G Magnetic Beads (Cat# P2106; Beyotime, China) for another 6 hours. The magnetic beads were washed 3 times with TBST. After the last washing, the proteins were eluted with elution buffer and subjected to western blot.

2.10 Transwell assay

Transwell assays were performed as previously described[16]. Transwell invasion assay was performed using 8.0 μm Transwell Permeable Supports (Corning, USA). BMDMs were resuspended in 100 μL of serum-free medium and seeded at a density of 1×10^5 cells into the upper chamber. The lower chamber was concurrently filled with 600 μL of medium containing 10% FBS. Following incubation, non-invading cells were mechanically removed from the membrane using a cotton swab. The invaded cells on the bottom surface of the membrane were fixed with 4% paraformaldehyde for 10 minutes and then stained with a 0.4% crystal violet solution. The migrated cells were visualized and imaged using a digital microscope.

2.11 In Situ Dihydroethidium (DHE) Staining

Superoxide generation was assessed using in situ dihydroethidium (DHE) staining (Cat# S0063; Beyotime, China)[17]. Fresh AAA specimens were promptly embedded in OCT and sectioned into frozen sections with a thickness of 8 μm. Subsequently, cryosections were incubated with DHE at 37°C for approximately 30 minutes. Images were captured under an Eclipse TE2000-U fluorescence microscope (Nikon, Japan).

2.12 Assay of Matrix Metalloproteinase Activity

To assess the activity of matrix metalloproteinases in AAA, an in situ zymogram detection method was conducted following the manufacturer's protocol (Cat# GMS80062.1; Genmed Scientifics Inc, Oxford, UK) as described previously[18]. FITC-labeled gelatin was utilized as a substrate to analyze the degradation activity of gelatin in non-fixed frozen tissue sections. The slides were incubated at room temperature in the absence of light for a duration of 2 hours. The activity of protein hydrolase was subsequently detected as green fluorescence using a fluorescence microscope (Leica DM3000B; Germany).

2.13 RNA-sequencing

Total aortic tissue RNA was extracted using Trizol reagent (Cat# 15596026; Life technologies,

California, USA). A total amount of 1 μg RNA per sample was used as input material for the RNA sample preparations. Sequencing libraries were generated using Hieff NGS Ultima Dual-mode mRNA Library Prep Kit for Illumina (Yeasen Biotechnology (Shanghai) Co., Ltd.) following manufacturer's recommendations. The libraries were sequenced on an Illumina NovaSeq6000 platform (Illumina, San Diego, CA, USA) to generate 150 bp paired-end reads, according to the manufacturer's instructions. The raw reads were further processed with a bioinformatic pipeline tool, BMKCloud (www.biocloud.net) online platform. Differential expression analysis of two groups was performed using the DESeq2. DESeq2 provide statistical routines for determining differential expression in digital gene expression data using a model based on the negative binomial distribution. The resulting P values were adjusted using the Benjamini and Hochberg's approach for controlling the false discovery rate. Genes with an adjusted P-value < 0.01 and Fold Change ≥ 2 found by DESeq2 were assigned as differentially expressed.

2.14 Detection of ROS

The concentration of reactive oxygen species (ROS) was assessed using an ROS kit (Cat# S003S; Beyotime, China)[19]. Dichlorodihydrofluorescein diacetate (DCFH-DA) was diluted in serum-free medium at a ratio of 1:1000 to achieve a final concentration of 10 μmol/L. The cell culture medium was removed and replaced with the diluted probe. The cells were incubated at 37 °C for 20 minutes. Following incubation, the medium was aspirated and the cells were washed three times with serum-free culture medium before being examined under a fluorescence microscope.

2.15 Measurement of cell viability

Cell viability was determined with Cell Counting Kit-8 (CCK-8) assays (Dojindo, Kumamoto, Japan) as described previously[20]. BMDMs were seeded into 96-well plates at a density of approximately 5000 cells per well and cultured for a designated period of time. 10 μL CCK-8 reagent was added to 100 μL of fresh medium per well and incubated for 1 hour at 37 °C. The optical density (OD) value at 450 nm was measured using microplate spectrophotometer.

2.16 Measurement of GSH/GSSG

The reduced glutathione (GSH) and glutathione disulfide (oxidized glutathione, GSSG) were evaluated by a GSH and GSSG Assay Kit (Cat# S0053; Beyotime; China), according to the manufacturer's instructions[21]. The ratio of GSH to GSSG (GSH/GSSG) was subsequently calculated.

2.17 Measurement of malondialdehyde (MDA)

Malondialdehyde (MDA) is a major indicator of lipid peroxidation, which was assessed with a Lipid Peroxidation MDA Assay Kit (Cat# S0131S; Beyotime; China), according to the manufacturer's instructions[22]. Briefly, the samples were lysed in RIPA buffer and then centrifuged at 12,000 g for 15 minutes. An aliquot (100 μL) of each cell supernatant was then reacted with the MDA working solution, which comprised thiobarbituric acid (TBA) diluent, TBA storage solution, and antioxidants. Following a 15-minute incubation at 100°C, the reaction mixtures were cooled to room temperature in a water bath and centrifuged at 1,000 g for 10 minutes. The absorbance of the resulting supernatant (100 μL) was measured at 532 nm.

2.18 Measurement of ferrous iron (Fe^{2+})

The relative iron concentration in cell lysates was assessed with an Iron Assay Kit (Cat# ab83366; Abcam) according to the manufacturer's instructions[23]. Briefly, the cells were collected and homogenized with iron assay buffer (100 μL) on ice. The samples were then centrifuged at 16,000 g for 10 minutes at 4°C. The supernatant was collected. Iron reducer (5 μL) was added, mixed, and incubated at 37 °C for 30 minutes. A volume of 100 μL iron probes was then added and mixed thoroughly. The reaction mixture was incubated at 37 °C for 60 minutes. The absorbance was measured on a colorimetric microplate reader at 593 nm.

2.19 Measurement of FerroOrange

A FerroOrange probe (Cat# F374; Dojindo, Shanghai, China) was used to detect intracellular Fe^{2+} as described previously[24]. After the indicated treatments, cells were incubated with FerroOrange working solution (1 μmol/L) for 30 minutes, and images were captured using a fluorescence microscope at excitation of 543 nm and emission of 580 nm.

2.20 Measurement of Mito-FerroGreen

A Mito-FerroGreen probe (Cat# M489; Dojindo, Shanghai, China) was used to detect mitochondrial Fe^{2+} as described previously[24]. After the indicated treatments, cells were incubated with Mito-FerroGreen working solution (5 μmol/L) for 30 minutes, and images were captured using a fluorescence microscope at excitation of 505 nm and emission of 535 nm.

2.21 Vascular ultrasound imaging

Vascular ultrasound was performed using a Vevo 3100 Imaging System (FUJIFILM VisualSonics, Toronto, ON, Canada) as described previously[18, 25]. Briefly, abdominal hair was removed with depilatory cream, and ultrasound gel was applied for clear imaging. During the imaging, the mice are anesthetized using 1.5%-2.0% isoflurane. Heart rate and respiration were monitored throughout. The abdominal aorta was visualized in brightness (B)-mode. The probe was placed transversely to locate the abdominal aorta, using the "Portal Triad" (comprising the hepatic artery, hepatic vein, and bile duct) as anatomical markers. The probe was first centered on the aorta, then moved down to find the kidney and adjusted to the long axis. For a longitudinal view of the abdominal aorta, the probe was aligned parallel to it, and images were taken to measure the aortic diameter.

2.22 Immunoprecipitation-coupled mass spectrometry (IP-MS)

Immunoprecipitation coupled to mass spectrometry was used to identify the binding partners of SENP3 in 293T cells. 293T cells were transfected with Flag-SENP3 or Flag-pcDNA for 48 hours. Transfected cells were lysed in cold lysis buffer (Cat# P0013; Beyotime) on ice for 30 minutes, followed by centrifugation at 12,000 rpm for 15 minutes. Cell lysates were immunoprecipitated with anti-Flag magnetic beads, as described above. Immunoprecipitated proteins were eluted with SDS sample buffer and analyzed on a PAGE gel, followed by liquid chromatography–tandem mass spectrometry (LC–MS/MS). Each sample underwent three LC-MS/MS runs on a Fusion quadrupole-Orbitrap mass spectrometer with an Easy-nLC 1200 UPLC system and Nanospray Flex ion source. Proteins were identified using MaxQuant v1.6.2.10 against the Uniprot Human and Mouse database and analyzed in Perseus 1.6.12.0.

2.23 Transmission Electron Microscopy

Morphological abnormalities in the mitochondria of bone marrow-derived macrophages (BMDMs) were identified using transmission electron microscopy (TEM). The BMDMs were initially fixed with 2.5% glutaraldehyde, followed by staining with cacodylate-buffered osmium tetroxide, and subsequently embedded in epoxy resin. Ultrathin sections were then prepared and analyzed with an electron microscope (Philips CM10, Philips, Eindhoven, Netherlands).

2.24 Morphological observation of mitochondria by confocal microscopy

Mito-Tracker (Invitrogen, USA) staining was employed to reveal the morphology of mitochondria in BMDMs treated with 1 μmol/L AngII for 24 hours, according to the instruction manual. Fluorescence images of mitochondria were obtained by confocal microscopy (FLUOVIEW FV3000; Olympus, Tokyo, Japan).

2.25 Berlin blue staining

Berlin blue staining (Cat# 60533ES20; Yeasen, China) was performed according to manufacturer's instructions. Briefly, potassium ferrocyanide is employed to isolate trivalent iron ions from proteins, resulting in a reaction that produces a blue ferric ferrocyanide precipitate, which is insoluble in water.

2.26 Measurement of H_2S

The concentration of H_2S was quantified spectrophotometrically employing the methylene blue method according to the kit instruction (Cat# A146-1; Nanjing jiancheng Bioengineering, China)[26]. Briefly, H_2S reacts with zinc acetate, N,N-dimethyl-p-phenylenediamine and ammonium ferric sulfate to generate methylene blue. Methylene blue has a maximum absorption peak at 665nm. The H_2S content can be calculated by measuring its absorbance value.

Reference

1. Satoh K, Nigro P, Matoba T, O'Dell MR, Cui Z, Shi X, Mohan A, Yan C, Abe J, Illig KA and Berk BC. Cyclophilin A enhances vascular oxidative stress and the development of angiotensin II-induced aortic aneurysms. *Nat Med*. 2009;15:649-56.
2. Salloum FN, Chau VQ, Hoke NN, Abbate A, Varma A, Ockaili RA, Toldo S and Kukreja RC. Phosphodiesterase-5 inhibitor, tadalafil, protects against myocardial ischemia/reperfusion through protein-kinase g-dependent generation of hydrogen sulfide. *Circulation*. 2009;120:S31-6.
3. Wang K, Ahmad S, Cai M, Rennie J, Fujisawa T, Crispi F, Baily J, Miller MR, Cudmore M, Hadoke PW, Wang R, Gratacós E, Buhimschi IA, Buhimschi CS and Ahmed A. Dysregulation of hydrogen sulfide producing enzyme cystathionine γ-lyase contributes to maternal hypertension and placental abnormalities in preeclampsia. *Circulation*. 2013;127:2514-22.
4. Campolo M, Esposito E, Ahmad A, Di Paola R, Wallace JL and Cuzzocrea S. A hydrogen sulfide-releasing cyclooxygenase inhibitor markedly accelerates recovery from experimental spinal cord injury. *Faseb j*. 2013;27:4489-99.
5. Van Dingenen J, Pieters L, Vral A and Lefebvre RA. The H(2)S-Releasing Naproxen Derivative ATB-346 and the Slow-Release H(2)S Donor GYY4137 Reduce Intestinal Inflammation and Restore Transit in Postoperative Ileus. *Front Pharmacol*. 2019;10:116.
6. Campolo M, Esposito E, Ahmad A, Di Paola R, Paterniti I, Cordaro M, Bruschetta G, Wallace JL and Cuzzocrea S. Hydrogen sulfide-releasing cyclooxygenase inhibitor ATB-346 enhances motor function and reduces cortical lesion volume following traumatic brain injury in mice. *J Neuroinflammation*. 2014;11:196.
7. Zhou Q, Zhang Y, Wang B, Zhou W, Bi Y, Huai W, Chen X, Chen Y, Liu Z, Liu X and Zhan Z. KDM2B promotes IL-6 production and inflammatory responses through Brg1-mediated chromatin remodeling. *Cell Mol Immunol*. 2020;17:834-842.
8. Tian D, Teng X, Jin S, Chen Y, Xue H, Xiao L and Wu Y. Endogenous hydrogen sulfide improves vascular remodeling through PPARδ/SOCS3 signaling. *J Adv Res*. 2021;27:115-125.
9. Tian D, Dong J, Jin S, Teng X and Wu Y. Endogenous hydrogen sulfide-mediated MAPK inhibition preserves endothelial function through TXNIP signaling. *Free Radic Biol Med*. 2017;110:291-299.
10. De Cicco P, Panza E, Ercolano G, Armogida C, Sessa G, Pirozzi G, Cirino G, Wallace JL and Ianaro A. ATB-346, a novel hydrogen sulfide-releasing anti-inflammatory drug, induces apoptosis of human melanoma cells and inhibits melanoma development in vivo. *Pharmacol Res*. 2016;114:67-73.
11. Huang C, Han Y, Wang Y, Sun X, Yan S, Yeh ET, Chen Y, Cang H, Li H, Shi G, Cheng J, Tang X and Yi J. SENP3 is responsible for HIF-1 transactivation under mild oxidative stress via p300 de-SUMOylation. *Embo j*. 2009;28:2748-62.
12. Zhou Z, Wang M, Li J, Xiao M, Chin YE, Cheng J, Yeh ET, Yang J and Yi J. SUMOylation and SENP3 regulate STAT3 activation in head and neck cancer. *Oncogene*. 2016;35:5826-5838.
13. Liang M, Cai Z, Jiang Y, Huo H, Shen L and He B. SENP2 Promotes VSMC Phenotypic Switching via Myocardin De-SUMOylation. *Int J Mol Sci*. 2022;23.
14. Cai Z, Wang Z, Yuan R, Cui M, Lao Y, Wang Y, Nie P, Shen L, Yi J and He B. Redox-sensitive enzyme SENP3 mediates vascular remodeling via de-SUMOylation of beta-catenin and regulation of its stability. *EBioMedicine*. 2021;67:103386.
15. Jiang Y, Liang M, Chen L, Wang J, Huang Y, Huo H, Xiao D, Hu Y, Wang Z, Ji Q, Li Y, Cai Z and He B. Myeloid SENP3 deficiency protects mice from diet and age-induced obesity via regulation of YAP1 SUMOylation. *Cell Mol Life Sci*. 2023;81:4.

16. Lu W, Zhang H, Niu Y, Wu Y, Sun W, Li H, Kong J, Ding K, Shen HM, Wu H, Xia D and Wu Y. Long non-coding RNA linc00673 regulated non-small cell lung cancer proliferation, migration, invasion and epithelial mesenchymal transition by sponging miR-150-5p. *Mol Cancer*. 2017;16:118.
17. He Z, Zhang X, Chen C, Wen Z, Hoopes SL, Zeldin DC and Wang DW. Cardiomyocyte-specific expression of CYP2J2 prevents development of cardiac remodelling induced by angiotensin II. *Cardiovasc Res*. 2015;105:304-17.
18. Sun LY, Lyu YY, Zhang HY, Shen Z, Lin GQ, Geng N, Wang YL, Huang L, Feng ZH, Guo X, Lin N, Ding S, Yuan AC, Zhang L, Qian K and Pu J. Nuclear Receptor NR1D1 Regulates Abdominal Aortic Aneurysm Development by Targeting the Mitochondrial Tricarboxylic Acid Cycle Enzyme Aconitase-2. *Circulation*. 2022;146:1591-1609.
19. Lei Y, Wang Y, Shen J, Cai Z, Zhao C, Chen H, Luo X, Hu N, Cui W and Huang W. Injectable hydrogel microspheres with self-renewable hydration layers alleviate osteoarthritis. *Sci Adv*. 2022;8:eabl6449.
20. Zhang Q, Lou Y, Zhang J, Fu Q, Wei T, Sun X, Chen Q, Yang J, Bai X and Liang T. Hypoxia-inducible factor-2α promotes tumor progression and has crosstalk with Wnt/β-catenin signaling in pancreatic cancer. *Mol Cancer*. 2017;16:119.
21. Li C, Chen X, Zhang S, Liang C, Ma X, Zhang R and Yan H. Glutaredoxin 1 protects lens epithelial cells from epithelial-mesenchymal transition by preventing casein kinase 1α S-glutathionylation during posterior capsular opacification. *Redox Biol*. 2023;62:102676.
22. Li N, Yi X, He Y, Huo B, Chen Y, Zhang Z, Wang Q, Li Y, Zhong X, Li R, Zhu XH, Fang Z, Wei X and Jiang DS. Targeting Ferroptosis as a Novel Approach to Alleviate Aortic Dissection. *Int J Biol Sci*. 2022;18:4118-4134.
23. Chen L, Liu Y, Wang Z, Zhang L, Xu Y, Li Y, Zhang L, Wang G, Yang S and Xue G. Mesenchymal stem cell-derived extracellular vesicles protect against abdominal aortic aneurysm formation by inhibiting NET-induced ferroptosis. *Exp Mol Med*. 2023;55:939-951.
24. Yu F, Zhang Q, Liu H, Liu J, Yang S, Luo X, Liu W, Zheng H, Liu Q, Cui Y, Chen G, Li Y, Huang X, Yan X, Zhou J and Chen Q. Dynamic O-GlcNAcylation coordinates ferritinophagy and mitophagy to activate ferroptosis. *Cell Discov*. 2022;8:40.
25. Cai Z, Satyanarayana G, Song P, Zhao F, You S, Liu Z, Mu J, Ding Y, He B and Zou MH. Regulation of Ptbp1-controlled alternative splicing of pyruvate kinase muscle by Liver kinase b1 governs vascular smooth muscle cell plasticity in vivo. *Cardiovasc Res*. 2024.
26. Yang G and Qiu Y. Effects of amlodipine combined with atorvastatin on Th17/Treg imbalance and vascular microcirculation in hypertensive patients with atherosclerosis: A double-blind, single-center randomized controlled trial. *Medicine (Baltimore)*. 2023;102:e32384.

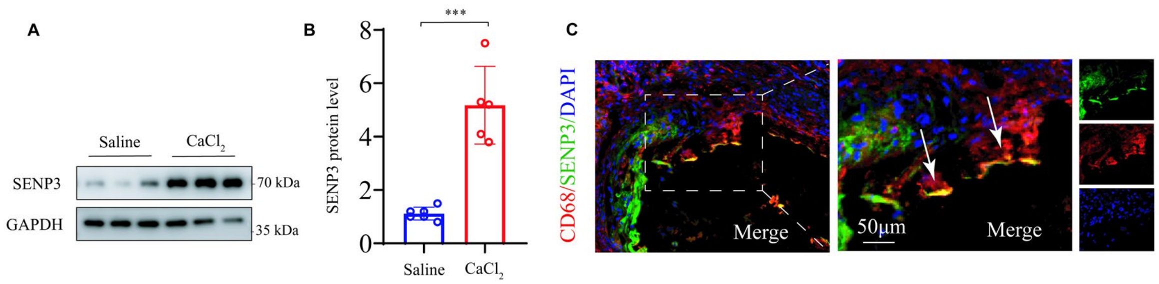

Figure S1. SENP3 is upregulated in aneurysmal aortas from $CaCl_2$-treated C57BL/6J mice.
A and **B,** SENP3 protein levels were determined by western blot in abdominal aortic samples from $CaCl_2$-treated C57BL/6J mice and control mice. Data represent mean ± SEM. P values were determined using student's t-test (B). ***$p < 0.001$. **C.** Representative images of dual immunofluorescence staining of SENP3 (green) and CD68 (red) in abdominal aortic samples from $CaCl_2$-treated C57BL/6J mice. Where indicated, nuclei were counterstained with DAPI (blue). Scale bar: 50 μm.

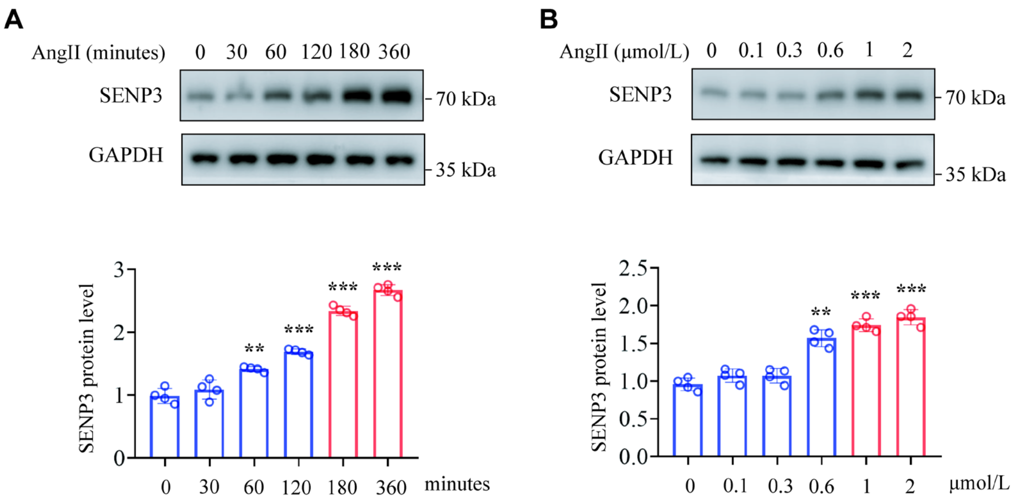

Figure S2. AngII markedly increased SENP3 protein expression in human THP-1-derived macrophages.

A and **B,** SENP3 protein levels were determined by western blot in THP-1-derived macrophages after stimulation with AngII (1 μmol/L) for the indicated time periods (A) or the indicated concentrations of AngII for 180 minutes (B) (n = 4 per group). Data represent mean ± SEM. P values were determined using Welch ANOVA test (A and B). For all panels, $*p < 0.05$; $**p < 0.01$; $***p < 0.001$.

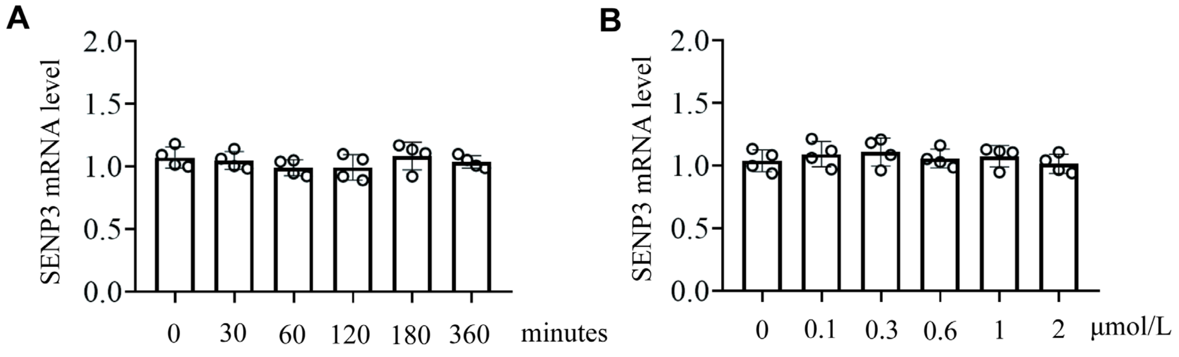

Figure S3. No significant change of SENP3 mRNA level in BMDMs after AngII stimulation.
A and **B,** The relative mRNA levels of SENP3 were determined by real-time quantitative polymerase chain reaction (RT-qPCR) in BMDMs after stimulation with AngII (1 µmol/L) for the indicated time periods (A) or with indicated concentrations of AngII for 180 minutes (B). Data represent mean ± SEM. P values were determined using Welch ANOVA test (A and B). For all panels, *p < 0.05; **p < 0.01; ***p < 0.001.

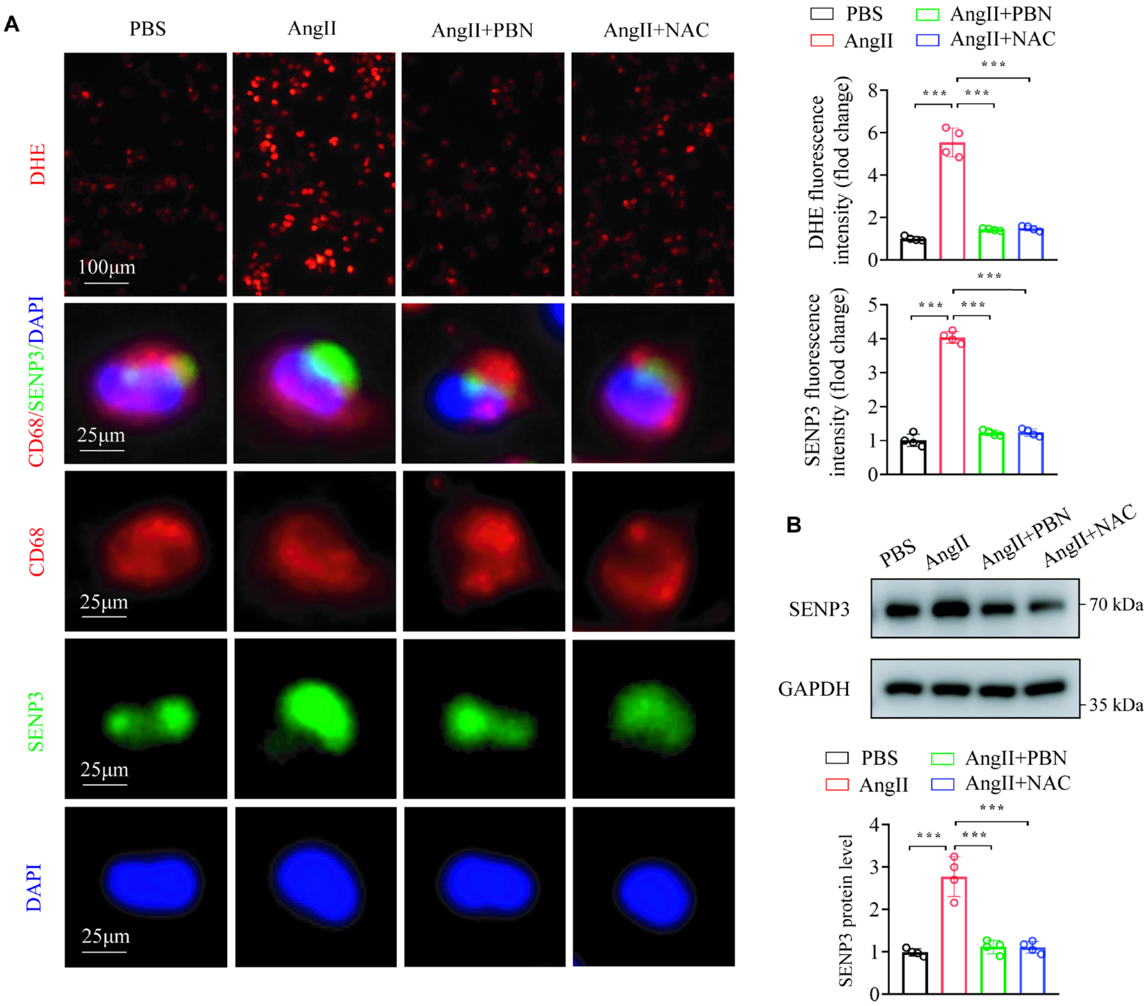

Figure S4. SENP3 is upregulated by in BMDMs in a ROS-dependent manner.

BMDMs were pretreated either PBN (10 mmol/L) or NAC (10 mmol/L) for 60 minutes, and were then subjected to AngII (1 μmol/L) or PBS treatment for another 180 minutes. **A,** Upper panel: Representative images of in situ DHE staining in the indicated groups; Lower panel: Representative images of dual immunofluorescence staining for SENP3 (green) and CD68 (red) in BMDMs from the indicated groups. Where indicated, nuclei were counterstained with DAPI (blue). Scale bar: 100 μm and 25 μm, respectively. **B,** Protein expression of SENP3 was examined by western blot in the indicated groups. Data represent mean ± SEM. P values were determined using two-way ANOVA followed by Bonferroni post-hoc test (A and B). For all panels, ***$p < 0.001$.

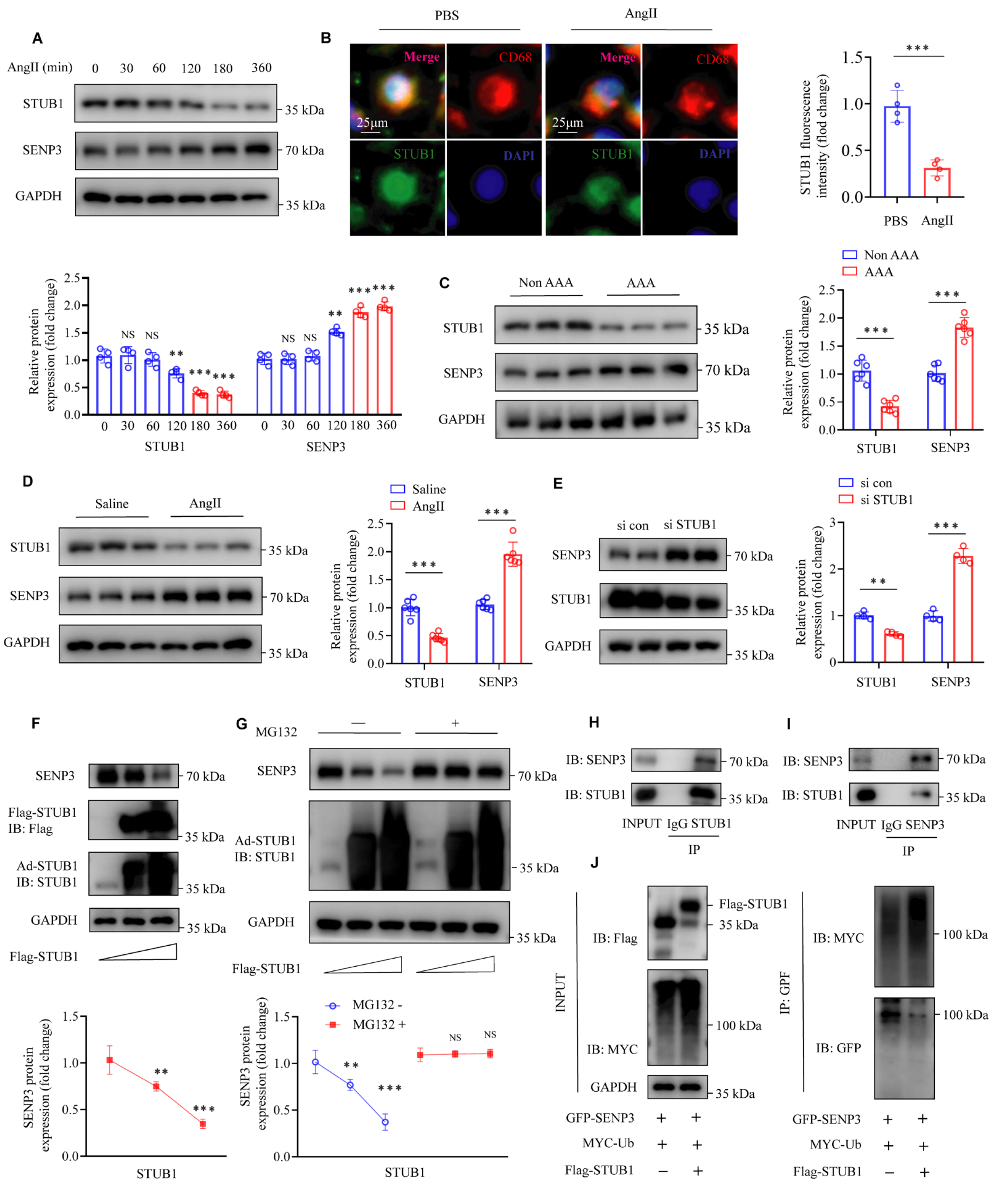

Figure S5. SENP3 expression is negatively regulated by the E3 ubiquitin ligase STUB1/CHIP. **A,** STUB1 protein levels were determined by western blot in BMDMs stimulated with AngII (1 μmol/L) for the indicated time periods. **B,** Representative images of dual immunofluorescence staining for STUB1 (green) and CD68 (red) in the BMDMs stimulated by AngII (1 μmol/L for 180 minutes). Where indicated, nuclei were counterstained with DAPI (blue). Scale bar: 25 μm. **C,** STUB1 and SENP3 protein levels were determined by western blot in human AAA samples and adjacent nonaneurysmal control samples (non-AAA) (n = 6 per group). **D,** STUB1 and SENP3 protein levels were determined by western blot in mouse abdominal aortic samples of *ApoE*$^{-/-}$ mice infused with AngII or saline for 28

days (n = 6 per group). **E,** BMDMs were transfected with control siRNA (si-NC) and STUB1 siRNA (si-STUB1) for 48 hours. Lysates were prepared and analyzed by western blot. **F,** BMDMs were infected with increasing amounts of adenovirus expressing Flag-STUB1 for 48 hours. The levels of Flag-STUB1 and SENP3 in whole cell lysates were determined by western blot with anti-Flag, anti-STUB1, anti-SENP3, and anti-GAPDH antibodies. **G,** BMDMs were infected with increasing amounts of adenovirus expressing Flag-STUB1 for 36 hours, in the presence or absence of MG132 (10 μmol/L) for the last 10 hours. The levels of Flag-STUB1 and SENP3 in whole cell lysates were determined by western blot with anti-Flag, anti-STUB1, anti-SENP3, and anti-GAPDH antibodies. **H** and **I,** The endogenous interaction between STUB1 and SENP3 in BMDMs was determined by co-immunoprecipitation assay. **J,** 293T cells were transfected with GFP-SENP3, Myc-ubiquitin (Myc-Ub), and Flag-STUB1 and treated with MG132 (10 μmol/L) for the last 10 hours. The ubiquitination of GFP-SENP3 was determined by immunoprecipitation assay and western blotting using antibody against Myc. Data represent mean ± SEM. Data represent mean ± SEM. P values were determined using Welch ANOVA test (A, F, and G) and student's t-test (B-E). For all panels, $^{*}p < 0.05$; $^{**}p < 0.01$; $^{***}p < 0.001$; ns: not significant ($P > 0.05$).

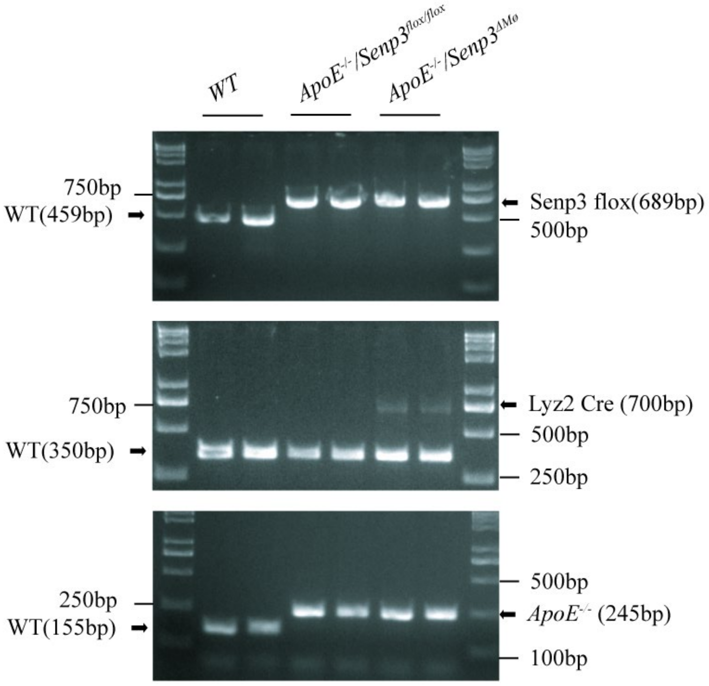

Figure S6. Generation of *ApoE$^{-/-}$;Senp3$^{flox/flox}$;Lyz2-Cre* mice. Genotype analysis of wild-type (WT), *ApoE$^{-/-}$;Senp3$^{flox/flox}$*, and *ApoE$^{-/-}$;Senp3$^{\Delta M\o}$* mice.

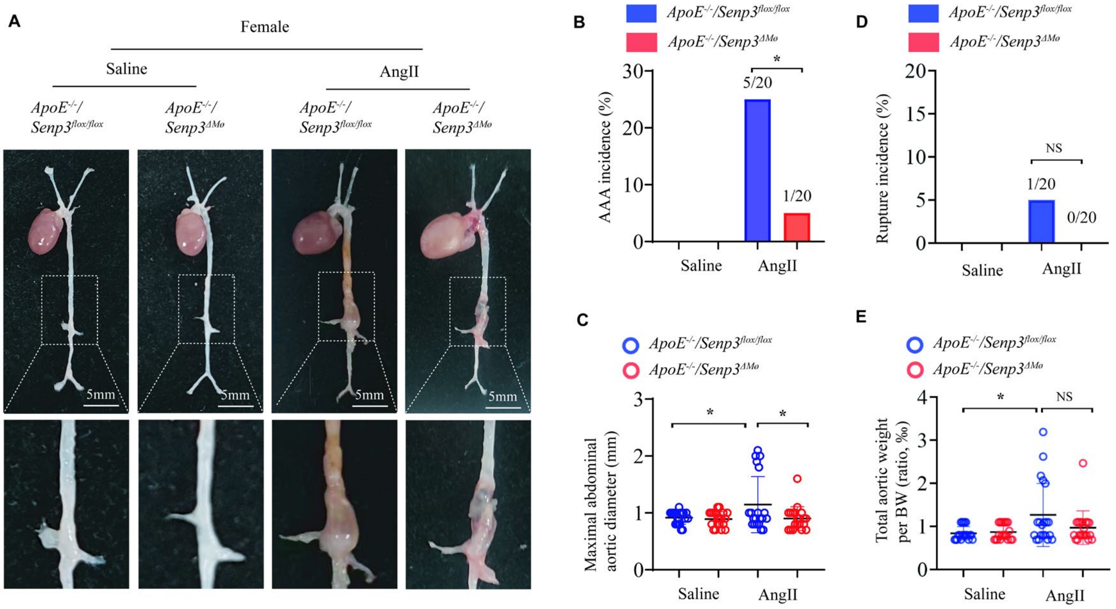

Figure S7. Myeloid-specific SENP3 deficiency blunts AngII-induced AAA formation in female mice.

Female *ApoE*$^{-/-}$*;Senp3*$^{flox/flox}$ and *ApoE*$^{-/-}$*;Senp3*$^{\Delta M\o}$ mice were subcutaneously injected with saline or AngII via a mini osmotic pump for 28 days (n = 20 per group). **A,** Representative images of the abdominal aorta visualized by macroscopic examination in the indicated groups. Scale bar: 5 mm. **B,** The incidence of AngII-induced AAA in the indicated groups. **C,** Quantification of the maximal abdominal aortic diameter measured by a digital vernier caliper in the indicated groups. **D,** The incidence of aortic rupture in the indicated groups. **E,** Quantification of the ratio of aortic weight to body weight (BW) in the indicated groups. Data represent mean ± SEM. P values were determined using Fisher exact test (B and D) and two-way ANOVA followed by Bonferroni post-hoc test (C and E).. For all panels, $*p < 0.05$; ns: not significant ($P > 0.05$).

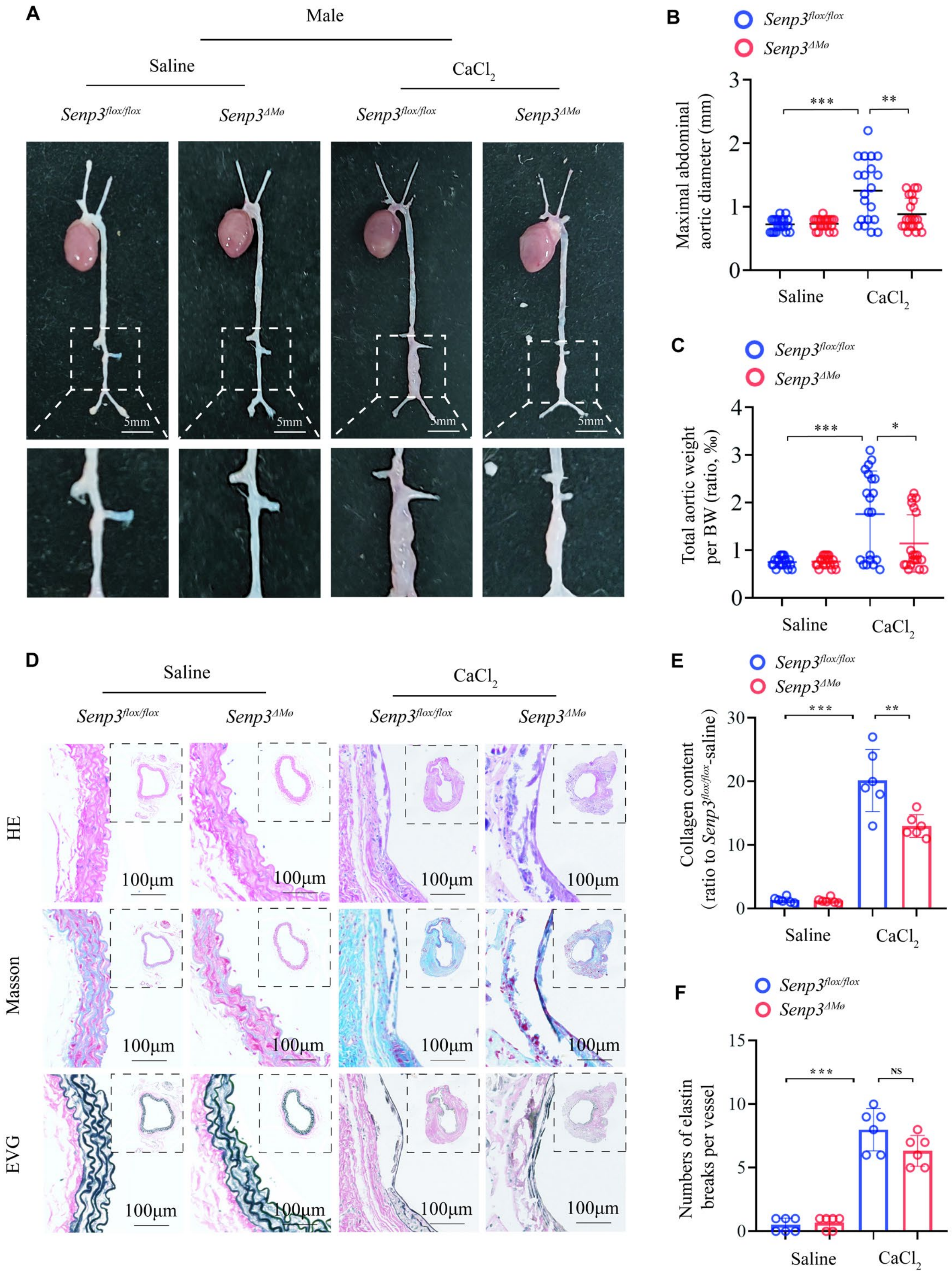

Figure S8. Myeloid-specific SENP3 deficiency attenuates $CaCl_2$-induced AAA formation.
A, Representative images of the macroscopic features of AAA induced by $CaCl_2$ in male *Senp3*$^{flox/flox}$ and *Senp3*$^{\Delta M\o}$ mice (n = 20 per group). Scale bar: 5 mm. **B** and **C,** Quantification of the maximal abdominal aortic diameter measured by a digital vernier caliper and ratio of aortic weight to body weight (BW) in the indicated groups. **D,** Representative images of infrarenal abdominal aortas sections stained with hematoxylin and eosin (H&E), Masson Trichrome, and Van Gieson. Scale bar: 100 μm. **E** and **F,** Quantification of relative collagen content (E) and numbers of elastin breaks per vessel (F) in the

indicated groups. Data represent mean ± SEM. P values were determined using two-way ANOVA followed by the Bonferroni post hoc test (B, C, E, and F). For all panels, *$p < 0.05$; **$p < 0.01$; ***$p < 0.001$; ns: not significant ($P > 0.05$).

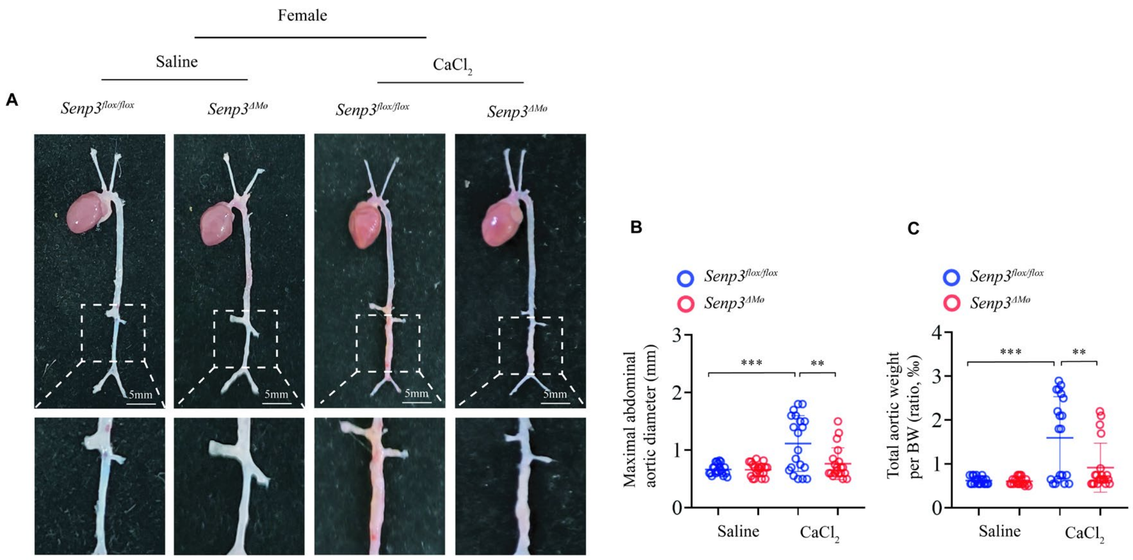

Figure S9. Myeloid-specific SENP3 deficiency restrains $CaCl_2$-induced AAA formation in female mice.

A, Representative images of the macroscopic features of AAA induced by $CaCl_2$ in female *Senp3*$^{flox/flox}$ and *Senp3*$^{\triangle M\o}$ mice (n = 20 per group). Scale bar: 5 mm. **B-C,** Quantification of the maximal abdominal aortic diameter measured by a digital vernier caliper and ratio of aortic weight to body weight (BW) in the indicated groups. Data represent mean ± SEM. Data were analyzed by two-way ANOVA followed by the Bonferroni post hoc test (B and C). For all panels, $^{**}p < 0.01$; $^{***}p < 0.001$.

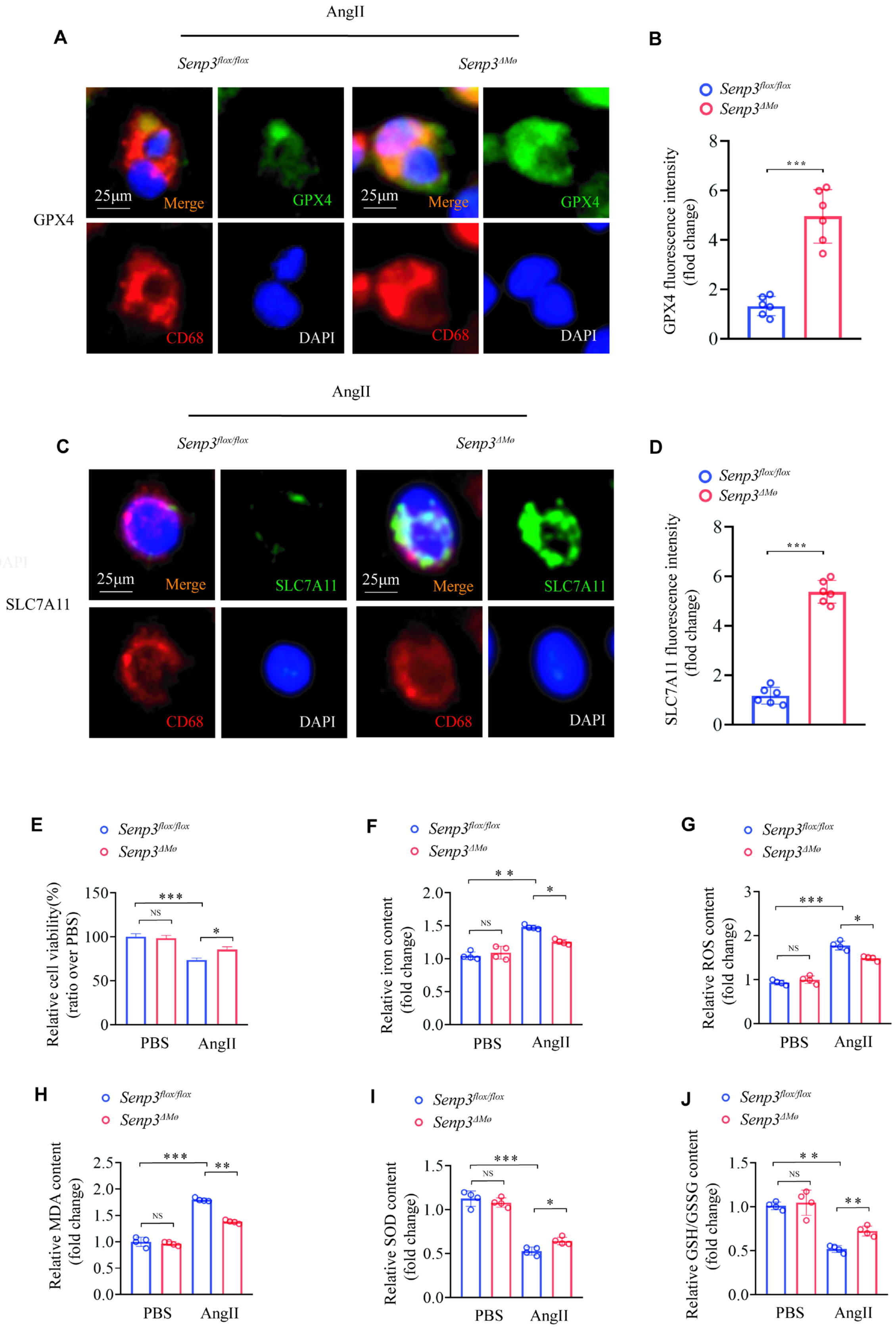

Figure S10. SENP3 regulates AngII-induced ferroptosis in BMDMs.

BMDMs isolated from *Senp3*$^{flox/flox}$ and *Senp3*$^{\triangle M\o}$ mice were stimulated with AngII (1μmol/L) or PBS for 24 hours. **A** and **B,** Representative images of dual immunofluorescence staining of GPX4 (green) and CD68 (red) in BMDMs in the indicated groups (A). Where indicated, nuclei were counterstained with DAPI (blue). Scale bar: 25 μm. Quantification of fluorescence intensity of GPX4 (B; n = 6 for each group). **C** and **D,** Representative images of dual immunofluorescence staining of SLC7A11 (green) and CD68 (red) in BMDMs in the indicated groups (C). Where indicated, nuclei were counterstained with DAPI (blue). Scale bar: 25 μm. Quantification of fluorescence intensity of SLC7A11 (D; n = 6 for each group). **E-J,** The relative cell viability, ferrous iron (Fe^{2+}) level, reactive oxygen species (ROS) generation, malondialdehyde (MDA) production, superoxide dismutase (SOD) level, and ratio of reduced glutathione (GSH) to oxidized glutathione (GSSG) (GSH/GSSG) in BMDMs in the indicated groups were measured. Data represent mean ± SEM. P values were determined using student's t-test (B and D) and two-way ANOVA followed by Bonferroni post-hoc test (E-J). For all panels, *p < 0.05; **p < 0.01; ***p < 0.001; ns: not significant (P > 0.05).

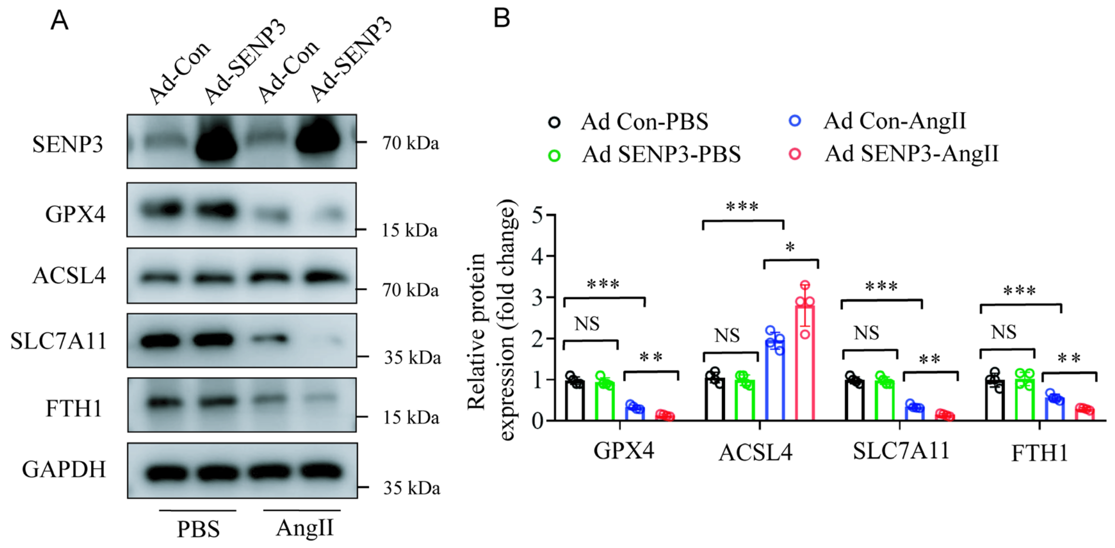

Figure S11: Overexpression of SENP3 exacerbates macrophage ferroptosis.

A and **B,** BMDMs were transfected with control adenovirus (Ad-con) and SENP3 adenovirus (Ad-SENP3) for 48 hours. The protein levels of GPX4, ACSL4, SLC7A11, and FTH1 were determined by western blot. Data represent mean ± SEM. P values were determined using two-way ANOVA followed by Bonferroni post-hoc test (B). For all panels, *p < 0.05; **p < 0.01; ***p < 0.001; ns: not significant (P > 0.05).

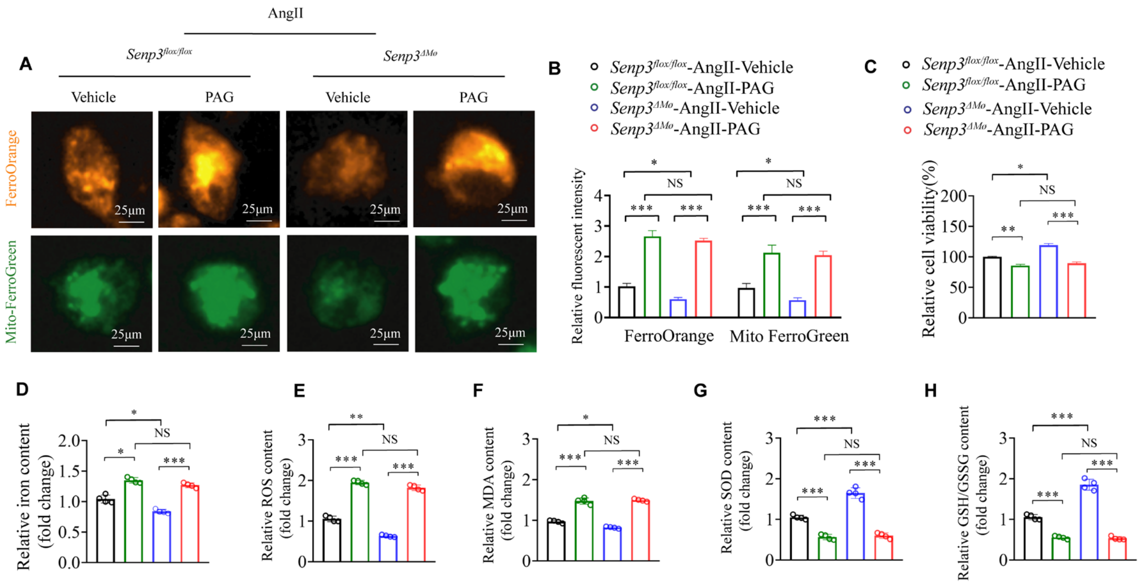

Figure S12. Pharmacological inhibition of CTH attenuates the protective effects of SENP3 deficiency on ferroptosis.

BMDMs isolated from *Senp3*$^{flox/flox}$ and *Senp3*$^{\triangle M\o}$ mice were pre-treated with PAG (1 mmol/L) for 60 minutes and were stimulated with AngII (1μmol/L) for another 24 hours. **A** and **B**, The levels of cellular ferrous iron (Fe^{2+}) and mitochondrial Fe^{2+} were assessed by FerroOrange and Mito-FerroGreen probes. Scale bar: 25 μm. **C-H,** The relative cell viability, Fe^{2+} level, ROS generation, MDA production, SOD level, and GSH/GSSG in BMDMs in the indicated groups were measured. Data represent mean ± SEM. P values were determined using two-way ANOVA followed by Bonferroni post-hoc test (B-H). For all panels, *p < 0.05; **p < 0.01; ***p < 0.001; ns: not significant (P > 0.05).

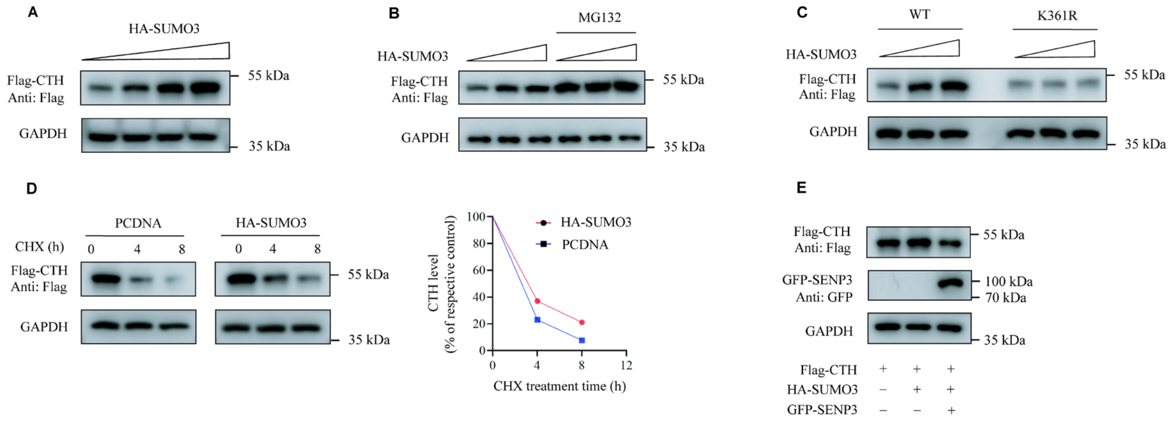

Figure S13. SUMO-3 promoted CTH protein stability.

A, 293T cells were transfected with Flag-CTH and increasing amounts of HA-SUMO3 for 48 hours. The protein levels of Flag-CTH, HA-SUMO3, and GAPDH in whole cell lysates were determined by western blot with anti-Flag, anti-HA and anti-GAPDH antibodies. **B,** 293T cells were transfected with Flag-CTH and increasing amounts of HA-SUMO3 for 48 hours, in the presence or absence of MG132 (10 μmol/L) for the last 10 hours. Lysates were prepared and analyzed by western blot. **C,** 293T cells were transfected with Flag-CTH/WT or Flag-CTH/K361R and increasing amounts of HA-SUMO3 for 48 hours. The levels of Flag-CTH in whole-cell lysates were determined by western blot with anti-Flag, anti-HA, and anti-GAPDH antibodies. **D,** 293T cells were transfected with Flag-CTH and PCDNA or HA-SUMO3 for 36 hours and were subsequently exposed to CHX for the indicated time. Lysates were prepared and analyzed by western blot. The relative level of CTH was evaluated by densitometry and normalized to GAPDH. **E,** 293T cells were transfected with Flag-CTH, HA-SUMO3 or GFP-SENP3 for 48 hours. Lysates were prepared and analyzed by western blot.

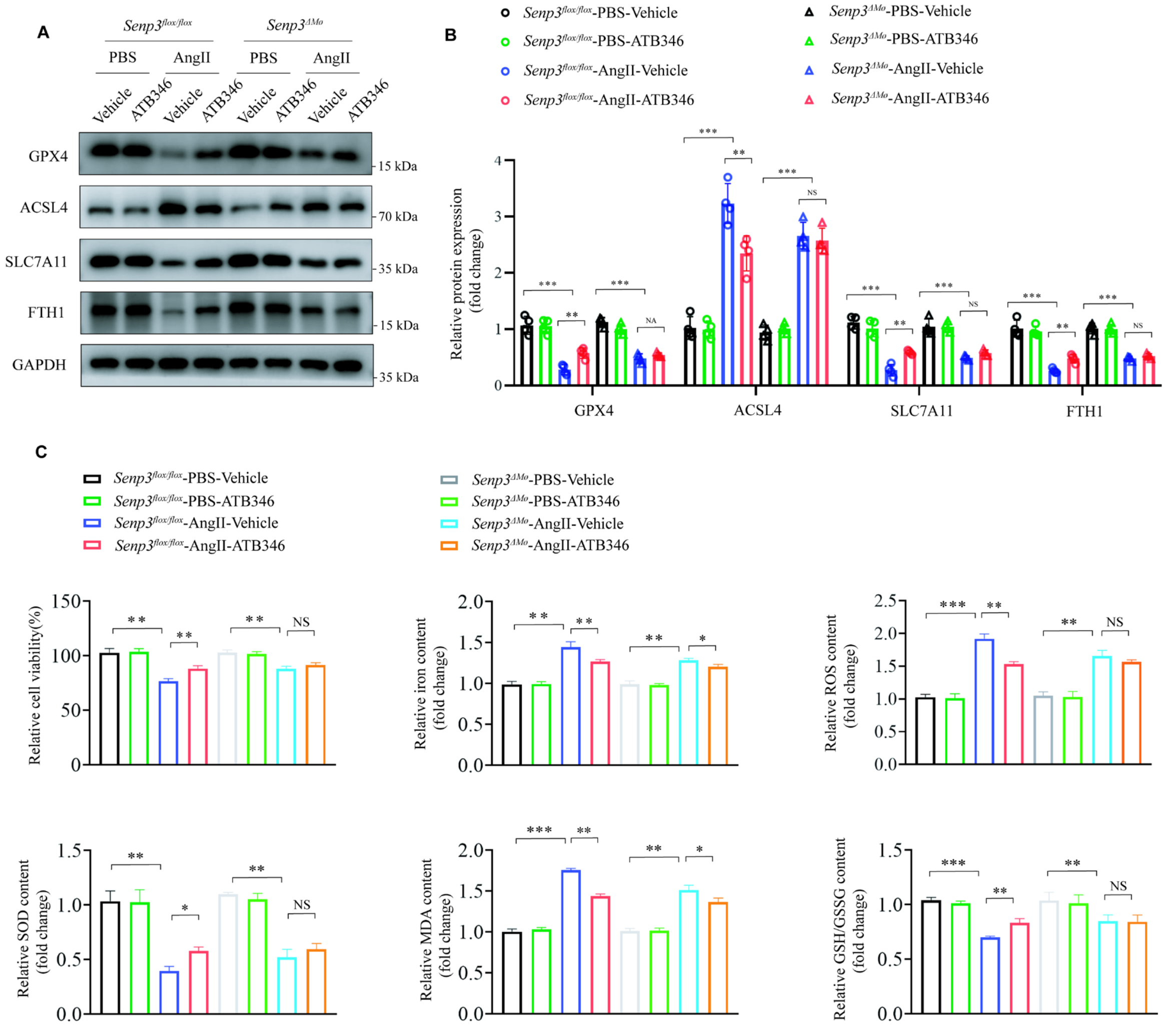

Figure S14. Supplementation with ATB346 alleviates AngII-induced ferroptosis in BMDMs. BMDMs isolated from *Senp3*$^{flox/flox}$ and *Senp3*$^{\Delta Mø}$ mice were pre-treated with ATB346 (100 μmol/L) for 60 minutes and were stimulated with AngII (1μmol/L) for another 24 hours. **A** and **B,** The protein levels of GPX4, ACSL4, SLC7A11 and FTH1 were determined by western blot in BMDMs in the indicated groups. **C,** The relative cell viability, Fe^{2+} level, ROS generation, MDA production, SOD level, and GSH/GSSG in BMDMs in the indicated groups were measured. Data were analyzed by two-way ANOVA followed by Bonferroni post-hoc test. *P<0.05; **P<0.01; ***P<0.001; ns: not significant (P > 0.05).

Table S1. The primer sequences for qPCR in this study.

Genes		Sequence (5'-3')
GAPDH (human)	Forward	AAGGTGAAGGTCGGAGTCAAC
GAPDH (human)	Reverse	CTTCCCGTTCTCAGCCATGTA
SENP3 (human)	Forward	ACCTCGCTGACATTCCACTG
SENP3 (human)	Reverse	CCCTTGCTGGGGTGAAAGAT
Gapdh (mouse)	Forward	AGGTCGGTGTGAACGGATTTG
Gapdh (mouse)	Reverse	TGTAGACCATGTAGTTGAGGTCA
Senp3 (mouse)	Forward	CTAGACGCGCTGAGGGTACT
Senp3 (mouse)	Reverse	TTTGAGTAAGTGGTGCCGGG
IL-1β (mouse)	Forward	GATGGCTGCACTATTCCTAATGCC
IL-1β (mouse)	Reverse	ATGGCTCTGAGAGACCTGACTTG
TNF-α (mouse)	Forward	TCCCAGGTTCTCTTCAAGGGA
TNF-α (mouse)	Reverse	GGTGAGGAGCACGTAGTCGG
CCL2 (mouse)	Forward	ACGCCCCACTCACCTGCTG
CCL2 (mouse)	Reverse	CCTGCTGCTGGTGATCCTCTTG
IL-6 (mouse)	Forward	GCCTTCTTGGGACTGATGCT
IL-6 (mouse)	Reverse	GGTCTGTTGGGAGTGGTATCC

Table S2. Si-RNA sequences.

Genes		Sequence (5'-3')
STUB1 (mouse)	Forward	GUGGCAGUGUACUACACUATT
STUB1 (mouse)	Reverse	UAGUGUAGUACACUGCCACTT
SENP3 (human)	Forward	GGAUGCUGCUCUACUCAAATT
SENP3 (human)	Reverse	UUUGAGUAGAGCAGCAUCCTT
CTH (mouse)	Forward	GGAUGGAGAAACAUUUCAATT
CTH (mouse)	Reverse	UUGAAAUGUUUCUCCAUCCTT
